# Glycyrrhizic acid combined with human adipose-derived MSCs synergistically alleviates the MPP+/MPTP-induced parkinson’s disease by inducing autophagy through PI3K/AKT/HIF-1α pathway

**DOI:** 10.1186/s13287-025-04626-6

**Published:** 2025-09-25

**Authors:** Xinlang Yu, Yuan Fang, Jiakang Zhang, Wenshan Li, Yajie Zhang, Qi Cui, Xiaoyu Liu, Yanjie Jiang, Qizhi Liu, Chengcheng Xu, Bin Jiang, Yan Lu

**Affiliations:** 1 Department of Brain Disease, Nanjing Hospital of Chinese Medicine, Nanjing University of Chinese Medicine, Nanjing, China; 2 Colorectal Surgery Center, Nanjing Hospital of Chinese Medicine, Nanjing University of Chinese Medicine, Nanjing, China; 3 Department of Gastroenterology, Nanjing Hospital of Chinese Medicine, Nanjing University of Chinese Medicine, Nanjing, China; 4 Center of Medicine Experiment, Nanjing Hospital of Chinese Medicine, Nanjing University of Chinese Medicine, Nanjing, China

**Keywords:** Glycyrrhizinic acid, Adipose-derived stem cells, Parkinson’s disease, PI3K/AKT/HIF-1α, Autophagy

## Abstract

**Aims:**

Emerging research highlights the considerable therapeutic promise of glycyrrhizic acid (GA) and stem cells used separately for Parkinson’s disease (PD). Nevertheless, the combined effects of GA and adipose-derived mesenchymal stem cells (ADSCs) in PD models have not been thoroughly investigated. This research is designed to evaluate the therapeutic potential of GA combined with ADSCs in vitro and in vivo, and to analyze the underlying molecular mechanisms.

**Main methods:**

In vitro experiments were performed in SH-SY5Y cell. In vivo experiments were performed in C57BL/6 mice.

**Key findings:**

In comparison to other treatment groups, the combination of GA and ADSCs exhibited improved therapeutic effects in vitro. RNA sequencing analysis revealed involvement of autophagy and the PI3K/AKT/HIF-1α signaling pathway in the treatment. In follow-up research, the combination of GA and ADSCs markedly increased the expression ratios of p-PI3K/PI3K, p-AKT/AKT, and p-mTOR/mTOR proteins in vitro following 1-methyl-4-phenylpyridinium (MPP+) exposure. Besides, the combined treatment downregulated the LC3II/LC3I expression ratio and Beclin-1 expression levels while upregulating p62, HIF-1α, and VEGFA expression levels. Similar to 3-MA, results from western blot, transmission electron microscopy (TEM) and immunofluorescence staining (IF) indicated that the combined treatment significantly reduced autophagy. However, treatment with the PI3K inhibitor (LY294002) reversed these increases in the expression ratios of p-PI3K/PI3K, p-AKT/AKT, and p-mTOR/mTOR, as well as the levels of p62, HIF-1α, and VEGFA. Moreover, LY294002 significantly impaired the autophagy-suppressing effects of the combination treatment. Finally, the combination of GA and ADSCs ameliorated behavioral deficits and pathological damage to dopaminergic neurons in PD mice induced by 1-methyl-4-phenyl-1,2,3,6-tetrahydropyridine (MPTP).

**Significance:**

The combination of GA and ADSCs displayed remarkable neuroprotective effects in vitro and in vivo. The underlying mechanism involves the regulation of autophagy via the PI3K/AKT/HIF-1α signaling pathway.

**Supplementary Information:**

The online version contains supplementary material available at 10.1186/s13287-025-04626-6.

## Introduction

Parkinson’s disease (PD) is a prevalent neurodegenerative disorder that impacts about 1% of those aged 60 and above [1]. This disease poses significant challenges to healthcare systems, especially in low- and middle-income countries such as Thailand [2]. PD is characterized by a range of motor symptoms, including tremor, rigidity, bradykinesia, and postural instability, in addition to non-motor symptoms that involve cognitive decline and mood disorders [3]. Pathologically, PD is defined by the progressive degeneration of dopaminergic neurons in the substantia nigra, accompanied by the accumulation of Lewy bodies—aggregates of misfolded α-synuclein protein [4]. This aggregation results in widespread cellular dysfunction, driven by mitochondrial impairment, oxidative stress, neuroinflammation and disruptions in the autophagy-lysosomal pathway, which together obstruct the removal of these toxic proteins [5]. Though levodopa reigns as the favored remedy for PD, effectively alleviating its symptoms, it remains powerless to impede the disease’s progression [6, 7]. Additionally, long-term administration of levodopa is linked to diminishing effectiveness and the onset of side effects, while non-motor symptoms remain largely unaddressed [8]. The shortcomings of current treatment options emphasize the pressing need for new strategies that target the fundamental pathophysiological mechanisms.

Glycyrrhizic acid (GA), a triterpenoid saponin derived from the root of Glycyrrhiza glabra, has been extensively investigated for its pharmacological properties [9]. As an inhibitor of high mobility group box 1 (HMGB1), a pro-inflammatory protein, GA shows therapeutic potential in HMGB1-mediated conditions such as traumatic brain injury, neuroinflammation, seizures, Alzheimer’s disease, multiple sclerosis and PD [10]. Research indicates that GA can mitigate oxidative stress and inhibit neuroinflammatory pathways, positioning it as a promising candidate for treating neurodegenerative diseases such as PD [11]. While GA has shown potential to induce autophagy in human breast cancer cells, the molecular mechanisms involved in the MPP+-induced PD model require further investigation [12].

Adipose-derived mesenchymal stem cells (ADSCs) are multipotent stem cells obtained from adipose tissue, characterized by their ability to self-renew and differentiate in multiple directions, offering promising therapeutic potential in regenerative medicine [13]. In the treatment of central nervous system (CNS) disorders that result in permanent neuronal damage, ADSC-based therapies—including transplantation and injections of exosomes or secretome can inhibit inflammatory responses, decrease neuronal apoptosis, support neural regeneration, and modulate immune functions [14–16]. A recent study showed that repeated intravenous administration of ADSCs in a mouse model of PD significantly improved motor function, protected dopaminergic neurons, and restored neurotrophic factor expression, underscoring their potential as a long-term therapeutic strategy for PD [17]. Another study further investigated the combination of Danhong injection (DHI) and mesenchymal stem cell (MSC) transplantation, finding that it significantly enhances MSC retention in cardiac tissue, improves cardiac function, and reduces myocardial infarct size compared to either treatment alone [18]. These results underscore the potential of Chinese medicine in advancing cell-based therapies. The neuroprotective effects of ADSCs and GA have led to investigations into their combination therapies [19]. We hypothesize that this integrated approach will deliver therapeutic outcomes superior to those achieved by using ADSCs or GA independently.

Autophagy is a crucial cellular process that maintains homeostasis by degrading damaged organelles and misfolded proteins. However, its role in neurodegenerative diseases, including PD, is complex and dualistic [20]. On one hand, autophagy serves as a critical cellular defense mechanism by facilitating the clearance of toxic protein aggregates, such as α-synuclein, thereby preventing neuronal apoptosis and maintaining cellular homeostasis. For instance, studies have demonstrated that the upregulation of autophagy significantly reduces the accumulation of α-synuclein aggregates, a pathological hallmark of PD, and attenuates the loss of dopaminergic neurons [21]. On the other hand, dysregulation of autophagy, particularly impaired autophagic flux, can exacerbate neuronal damage and contribute to cell death. In PD, defective autophagic activity results in the accumulation of cytotoxic protein aggregates and accelerates neurodegeneration [22]. Emerging evidence suggests that precise modulation of autophagy to restore its equilibrium may represent a promising therapeutic strategy to mitigate neurodegenerative processes and preserve dopaminergic neuronal integrity [23]. Notably, recent investigations have revealed that both GA and ADSCs possess the capacity to regulate autophagic activity, highlighting a potential mechanistic basis for their neuroprotective efficacy in PD models [24].

LC3-II, the form of LC3-I that binds to phosphatidylethanolamine, is associated with the autophagosome membrane, and its levels reflect the quantity of autophagosomes [25]. P62 accumulates and interacts with LC3B, reducing the aggresome signal. Mammalian target of rapamycin (mTOR), a serine/threonine kinase, acts as a negative regulator of autophagy by integrating signals from pathways that detect the cell’s energy state [26]. PI3K/AKT activation stimulates mTOR, influencing cell death via the regulation of LC3-I and LC3-II, thereby inhibiting autophagy [27]. Hypoxia-inducible factor 1α (HIF-1α), a crucial regulator of cellular responses to low oxygen levels, has been increasingly associated with PD. In PD, the activation of HIF-1α under hypoxic conditions enhances autophagy and facilitates α-synuclein aggregation, contributing to the rapid loss of dopaminergic neurons [28]. The latest research shows that GA can mitigate neuronal apoptosis by activating the PI3K/AKT signaling pathway [29], while HIF-1α further facilitates the activation of the AKT/mTOR pathway [30]. Under hypoxic conditions, ADSCs exhibit superior viability, significantly decreasing the expression of inflammatory factors and activating the PI3K/AKT/HIF-1α pathway. In this study, we investigated the combined effects of GA and ADSCs in MPP+-injured SY5Y cells and MPTP-induced PD model mice, evaluating their potential to mitigate PD via autophagy regulation.

## Materials and methods

### Animals and treatment

The work has been reported in line with the ARRIVE guidelines 2.0. Male C57BL/6 mice (8–10 weeks old, 20–22 g) were purchased from Shanghai SLAC Laboratory Animal Co., Ltd. (Shanghai, China) and all animals were maintained within specific pathogen-free facilities at the Laboratory Animal Center of Nanjing University of Chinese Medicine for the MPTP models. All animal treatments were approved by the Animal Ethics Committee of Nanjing University of Chinese Medicine (permit and approval number: A20241201) and performed in accordance with the institutional guidelines of Nanjing University of Chinese Medicine. Mice were housed in a controlled environment with a 12-hour light/dark cycle, maintained at a temperature of 22 ± 2 °C, and provided with food and water ad libitum. Surgical procedures were performed under isoflurane anesthesia. Mice were monitored daily for signs of pain or distress, and humane endpoints were strictly followed. According to the guidelines of the Animal Ethics Committee of Nanjing University of Chinese Medicine, mice were euthanized through cervical dislocation at the conclusion of the experiments. The MPTP-induced mouse model of PD was established as previously described and validated [31]. Briefly, mice were injected intraperitoneally with MPTP (30 mg/kg, MCE, China) continuously for a week to induce dopaminergic neuron degeneration. Control mice received an equivalent volume of saline. To ensure the reproducibility and reliability of the model, motor behavioral tests, including the open field test, rotarod test and pole test, were performed 7 days after the last MPTP injection to assess motor coordination and bradykinesia. After baseline behavioral assessments, animals were randomly assigned to the control (Con), model (Mod), GA, ADSCs, and GA + ADSCs groups. We used a total of 40 male C57BL/6 mice in five groups, with an average of 8 mice in each group. In the GA treatment group, GA (50 mg/kg, Shanghai Yuanye Bio-Technology, China) was administered simultaneously with MPTP modeling for 14 days. In the ADSCs group, ADSCs (1 × 10^6^ cells) were administered via tail vein injection once after the completion of MPTP modeling, with a second injection given on day 15. The in vivo protocol (ADSCs administration on day 1 and 15 post-MPTP) was based on literature and preliminary data. Intravenously delivered ADSCs exhibit limited persistence due to rapid clearance, but our pilot studies showed that a second injection significantly improved engraftment and coincided with peak motor deficits (day 15), ensuring cell presence during functional assessments. The timing aimed to target both acute neurotoxicity (day 1) and the therapeutic evaluation window (day 15), as cell signals peak within 24 h but decline rapidly [32–34]. In the GA + ADSCs group, a combination of GA treatment and ADSCs administration was employed.

### Open field test

The open field test was used to assess the spontaneous activity and exploratory behavior of mice. Mice were first placed in a rectangular arena (45 × 45 × 60 cm), and racking was started immediately by a computer tracking system. After acclimatization for 30 min, the movement trajectory of the animal was monitored by the tracking system. The arena was wiped with 75% alcohol and allowed to dry between trials.

### Pole test

The pole test, a widely used method for assessing motor coordination in models of movement disorders, was utilized in this study. The apparatus consisted of a base, a 60 cm rough-surfaced pole, and a cage box. Mice underwent training one day prior to the experiment. During the test, each mouse was placed head-up at the top of the pole, and the descent time to the base was recorded. Five trials were conducted per mouse, and the mean descent time was calculated. To minimize interference, the environment was kept quiet, and odors were eliminated with 75% alcohol before and after testing.

### Rotarod test

The rotarod test was conducted to assess motor coordination in mice by measuring the duration of their walking on a cylindrical rotating rotarod. A mouse fatigue rotarod apparatus was used, which gradually accelerated from 5 rpm to 30 rpm over a 5 min period. The time each mouse remained on the rotarod was recorded. Each mouse underwent at least three trials, with intervals of 30 min or longer between tests, and the average time from the three trials was calculated. During the test, the room was kept quiet, and the rotarod was cleaned with 75% alcohol to eliminate odors.

### Gait analysis

The CatWalk system was employed in this experiment to evaluate the natural motor function and coordination of mice. The system comprises a walking platform, a high-speed camera, backlight, a spring-loaded lift channel, and CatWalk^™^ XT 9.1 software. Prior to formal testing, mice underwent a 3-day training period, with at least five recordings per mouse. Testing was conducted in a dark and quiet environment. The walking platform was cleaned with 75% alcohol before and after each trial to eliminate debris and odors. Data were analyzed using CatWalk^™^ XT 9.1 software.

### Cell culture and identification

The neuroblastoma SH-SY5Y cell line was originally established by Biedler et al. and was obtained from the American Type Culture Collection (CRL-2266, ATCC, https://www.atcc.org) [35]. The adipose-derived mesenchymal stem cells (ADSCs) used in this study were kindly provided as a gift by Dr. Yuan Fang from Colorectal Surgery Center, Nanjing Hospital of Chinese Medicine. The ADSCs were isolated from human subcutaneous adipose tissue obtained during elective liposuction procedures, as previously described [36]. Briefly, adipose tissue was washed extensively with PBS to remove blood cells and debris, followed by enzymatic digestion with 0.1% collagenase type I at 37° C for 60 min. The digested tissue was then centrifuged to separate the stromal vascular fraction, which was resuspended in complete culture medium (DMEM supplemented with 10% fetal bovine serum and 1% penicillin/streptomycin). Cells were cultured in an incubator with humidified air containing 5% CO2 and 95% O2 in high-glucose Dulbecco’s modified Eagle’s medium (DMEM, Gibco, Thermo Fisher Scientific, USA) supplemented with 10% fetal bovine serum. Cells in the exponential growth phase were selected for subsequent experiments. SH-SY5Y cells were exposed to MPP+ (MCE, China) to induce neurotoxicity in vitro. After the MPP + treatment, glucose-free EBSS (with or without 3-methyladenine or LY294002) was added to replace the standard culture medium, and cells were incubated for 6 h. The MPP + treatment (with or without 3-methyladenine or LY294002) was terminated by switching from EBSS to the regular culture medium. Finally, the SY5Y cells were cocultured with GA, ADSCs, or both. GA was dissolved in distilled water to a final concentration of 100 µM. The final concentrations of 3-methyladenine and LY294002 were set to 10 µM and 10 mM, respectively.

### Coculture of SH-SY5Y cells with combined ADSCs and Glycyrrhizinic acid in a transwell system

SH-SY5Y cells were incubated with 1 mΜ MPP + for 24 h to establish cell model, after which cell viability was detected. A Transwell-6 system with a 0.4 μm porous membrane (Polyester or Polyethylene Terephthalate, PET, Corning^®^, Tewksbury, MA, USA) was used to prevent the transfer of vesicles larger than exosomes and to avoid direct cell contact for 24 h. They were then indirectly co-cultured with GA (at a concentration of 100 µM) alone or ADSCs (2 × 10^5^) alone or both in different groups (S1 Table). ADSCs were seeded in the upper chamber and SH-SY5Y cells were planted in the lower chamber. 3-MA (3-Methyladenine, a inhibitor of autophagy, MCE, China) and LY294002 (a inhibitor of PI3K, MCE, China) were used at a concentration of 10 mM and 10 µM. Before ADSCs were co-cultured with SH-SY5Y cells, they were stimulated with 3-MA or LY294002 for 6 h.

### Cell viability assay

The CCK-8 kit was assess to measure SH-SY5Y cell viability. Cells (5 × 10^4^/well) were seeded in 96-well plates and incubated overnight. To examine the effects of MPP + and GA on cell viability, cells were treated with varying concentrations of MPP+ (0, 0.5, 1, and 2 mM) for 24 h and GA (0, 3.125, 6.25, 12.5, 25, 50, 100, 150, and 200 µM) for 24–48 h. To test protective effects of GA, SH-SY5Y cells were pretreated with 1 mM MPP + for 24 h, followed by exposure to various GA concentrations (0, 3.125, 6.25, 12.5, 25, 50, 100, and 150 µM) for an additional 24 h. An MPP+-only group (1 mM) and a control group with normal media were included. At specified times, 100 µL of CCK-8 was added and incubated for 1–2 h. Optical density was measured at 450 nm using a microplate reader (Thermo Fisher Scientific, MA, USA). Each condition was tested in quadruplicate with three replicates per well, and viability was calculated relative to the control.

### ADSCs flow-cytometric analysis

Well-conditioned second-generation cells were expanded to the third generation, digested with trypsin, and counted. Cells were labeled with anti-CD73 (APC, Thermo Fisher Scientific, USA), CD90 (PE, Elabscience, China), CD105 (FITC, Thermo Fisher Scientific, USA), and anti-CD34, CD45 (PE, Elabscience, China). After incubation at 4 °C for 30 min, cells were washed twice with 1 mL PBS and resuspended in 500 µL of complete DMEM. Labeled cell intensity was measured via flow cytometry (Beckman, USA).

### Osteogenic, adipogenic, chondrogenic differentiation of ADSCs

The second-generation ADSCs were seeded in 6-well plates (Costar, Corning, USA) at a density of 2 × 10^4^ cells per well. Human Adipose-derived Mesenchymal Stem Cells Osteogenic Differentiation Kit (OriCell, China), Human Adipose-derived Mesenchymal Stem Cells Adipogenic Differentiation Kit (OriCell, China) and Human Adipose-derived Mesenchymal Stem Cells Chondrogenic Differentiation Kit (OriCell, China) were used used to verify the osteogenic, adipogenic and chondrogenic differentiation of ADSCs. Differentiation media were changed 2–3 day and induction until 21 days, cells were stained with oil red O, alcian blu and alizarin red staining, followed by microscopy observation [37].

### qRT-PCR assay

Total RNA was extracted by the FastPure Cell/Tissue Total RNA Isolation Kit V2 (Vazyme, China) and reverse transcribed into cDNA by HiScript III RT SuperMix for qPCR (+ gDNA wiper) (Vazyme, China). All primers were synthesized by GENEray Biotechnology (Shanghai, China), as shown in Table 1. On the Applied Biosystems 7500 Real-Time PCR System (Thermo Fisher Scientific, USA), gene expression was measured using ChamQ Universal SYBR qPCR Master Mix (Vazyme, China). The relative changes in candidate gene expression were analyzed using the 2-ΔΔCt method, with GAPDH serving as the internal reference.

**Table 1:** Primers sequence of all studied genes

mRNA	Primer sequence 5′−3′
PI3K	F: TTTTGCTGTTCGGTGCTTGG R: CCAAAAGCAGGCCAAACCTC
AKT	F: GCTACAAGGAACGGCCTCAG R: GTTTCCACATGGAAGGTGCG
mTOR	F: TGGAGAACCAGCCCATAAGAAA R: TGAGAGAAATCCCGACCAGTGA
TH	F: GGAAGGCCGTGCTAAACCT R: GGATTTTGGCTTCAAACGTCTC
SNCA	F: AAGAGGGTGTTCTCTATGTAGGC R: GCTCCTCCAACATTTGTCACTT
LRRK2	F: GAGCACGCCTCCAAGTTATTT R: ACTGGCATTATGAACTGTTAGCA
FOXA2	F: TGTTCGAGAACGGCTGCTAC R: CCCCGAGTTGAGCCTGTGAG
LMX1A	F: GCAAAGGGGACTATGAGAAGGA R: CGTTTGGGGCGCTTATGGT
NURR1	F: GTTCAGGCGCAGTATGGGTC R: CTCCCGAAGAGTGGTAACTGT
DAT	F: TTCCTCAACTCCCAGTGTGC R: AAGCCAATGACGGACAGGAG
MAP2	F: CGGAGTAACCAAGAGCCCAG R: ATAACTTGGTGGGGTGCCAG
TNF-α	F: CCTCTCTCTAATCAGCCCTCTG R: GAGGACCTGGGAGTAGATGAG
IL-1β	F: ATGATGGCTTATTACAGTGGCAA R: GTCGGAGATTCGTAGCTGGA
IL-6	F: ACTCACCTCTTCAGAACGAATTG R: CCATCTTTGGAAGGTTCAGGTTG
GAPDH	F: TGTTCGTCATGGGTGTGAAC R: ATGGCATGGACTGTGGTCAT

### Enzyme-linked immunosorbent assay (ELISA)

The cell supernatant was centrifuged at 3500 rpm for 10 min. Extract the serum and keep it in the refrigerator at −80 °C. The concentration of TNF-α, IL-1β, and IL-6 in the coculture medium were measured using a human specific enzyme-linked immunosorbent assay (ELISA) kit (MULTI SCIENCES BIOTECH, Wuhan, China) according to the manufacturer’s instructions. Absorbance measurements were taken with a microplate reader (Thermo Fisher Scientific, MA, USA) at a wavelength of 450 nm, and the results were compared to a standard curve.

### RNA sequencing and data analysis

SH-SY5Y cells were harvested from the culture plates in the Con, Mod, and GA + ADSCs groups (*n* = 3). RNA was lysed with Trizol reagent and quantified with T100 Thermal Cycler (BIO-RAD, California, USA). A sequencing library was constructed using MGISP-960 (MGI-TECH, Shenzhen, China), and qualified reads were mapped to the human reference genome. Differential expression analysis between groups was conducted using the DESeq2 R package. Genes were considered differentially expressed (DEGs) if they exhibited a log2 fold change ≥ 2 and a P value ≤ 0.05. Heat maps, principal component analysis (PCA), venn diagram and volcano plot were generated using the corresponding package. Gene Ontology (GO) enrichment and Kyoto Encyclopedia of Genes and Genomes (KEGG) pathway analysis were performed using the cluster Profiler R package.

### Western blot analysis

Proteins were extracted from SH-SY5Y cells or mice tissue using a mixture of RIPA buffer, phosphatase inhibitor, and protease inhibitor cocktail. After centrifugation at 12,000 rpm for 10 min at 4 °C, the protein concentration of the supernatant was determined using a bicinchoninic acid (BCA) protein assay kit (Epizyme Biotech, China). The protein concentration was then adjusted to 2 µg/µL and denatured by heating at 100 °C for 10 min. A total of 20 µg of protein per well was loaded onto an SDS-PAGE gel for electrophoresis, after which the proteins were transferred to a polyvinylidene fluoride (PVDF) membrane. PVDF membrane was incubated with specific primary antibodies including LC3B (1:1000, abcam), P62 (1:1000, GeneTex), Beclin-1 (1:1000, abcam), HIF-1α (1:1000, abcam), VEGFA (1:1000, abcam), p-PI3K (1: 1000, Abmart), p-AKT (1:1000, GeneTex), p-mTOR (1: 1000, CST), AKT (1: 1000, abcam), PI3K (1: 1000, CST), mTOR (1:1000, CST), TH (1:1000, GeneTex), α-Syn (1:1000, abcam), Nurr1 (1:1000, Proteintech), MAP2 (1:1000, Proteintech), FOXA2 (1:1000, Proteintech) and GAPDH (1:3000, CST) and secondary antibodies after being blocked for 1 h (Table. S2). The relative densities of the protein bands were detected by using the ChemiDoc XRS+ (Bio-Rad, USA).

### Immunofluorescence staining (IF)

After perfusion with saline to clear the heart, the entire mouse brain was meticulously extracted and placed in a 4% paraformaldehyde solution at 4 °C for fixation overnight. The tissue was then subjected to a stepwise dehydration process using a sucrose gradient, beginning with a 15% sucrose solution for 24 h, followed by a 30% sucrose solution for another 24 h, until the brain tissue sank to the bottom. Finally, the tissue was embedded in OCT and sectioned at a thickness of 30 μm. Every third brain Sect. (8 sections/mouse) with 30 μm thickness was obtained from bregma between AP − 2.80 and − 3.64 mm for immunofluorescence. The anatomical location of SNc was delineated according to the brain atlas (Paxinos atlas). For immunofluorescence analysis in cells, cells were seeded into a 24-well plate with glass coverslips (3 × 10^4^ per well). The sliced sections or treated SH-SY5Y cells were fixed with 4% paraformaldehyde, permeabilized with 0.25% Triton-X-100 in PBS and blocked with 1% bovine serum albumin, 10% normal goat serum, and 0.3% glycine in PBST for 2 h. Subsequently, the sliced sections or cells were incubated with anti-TH (Cell Signaling Technology, USA), anti-Nurr1 (Proteintech, China), anti-MAP2 (Proteintech, China), anti-FOXA2 (Proteintech, China) or anti-LC3B primary antibody (Cell Signaling Technology, USA) at 1:200 in a dark humidity box at 4 °C overnight, then incubated with a secondary antibody (Invitrogen, Alexa Fluor 488/594) goat anti-rabbit IgG (H + L) at 1:500 dilution for 2 h. Finally, after washing, the nuclei were stained with DAPI, and the cells were mounted for imaging. Fluorescence was observed using a fluorescence microscope with a 20× objective (final magnification 200×) to analyze TH, Nurr1, MAP2, FOXA2 or LC3B expression and localization in a double-blind manner by two operators (LEICA DMI3000B, Germany). Fluorescence intensities were analyzed by the ImageJ software.

### Transmission electron microscopy (TEM)

Ultrastructural changes in SH-SY5Y cells were evaluated using transmission electron microscopy (TEM). The cells were harvested and initially fixed with glutaraldehyde solution for 30 min, followed by centrifugation with 1% fetal bovine serum. The cells were then fixed overnight at 4 °C with 2.5% glutaraldehyde solution. Ultra-thin sections were stained with lead citrate and uranyl acetate. The samples were examined and photographed using a transmission electron microscope (TEM; H-7000; Hitachi, Tokyo, Japan).

### Immunohistochemistry

Following transcardial saline perfusion, whole brains were extracted and fixed in 4% PFA (light-protected, 48 h with agitation). Coronal Sect. (40 μm) spanning bregma − 2.80 to − 3.64 mm were processed through standard immunohistochemistry: dewaxing, antigen retrieval, peroxidase blocking, and incubation with primary (TH antibody) and secondary antibodies, followed by DAB development and hematoxylin counterstaining. For stereological quantification, we systematically sampled every third Sect. (8 sections/mouse) containing substantia nigra pars compacta (SNc), as identified using Paxinos atlas coordinates. Digital images were acquired using an Olympus VS120 slide scanner (20× objective, 3 Z-stacks at 5 μm intervals) for unbiased cell counting. Cell counting was performed and confirmed in a double-blind manner by two operators.

### Nissl and hematoxylin-eosin (H&E) staining

Nissl staining was performed to assess neuronal loss in both hippocampus and prefrontal cortex (PFC) following treatment. The frozen 18-µm-thick brain coronal slices of mice were soaked in Nissl staining solution for 5–15 min and then decolorized in 75, 80, 95, and 100% ethanol for 2 min each. Then the slices were sealed and observed under a microscope. Nissl-positive cells were counted with ImageJ software under 20× magnification. The major organs (i.e., heart, liver, lung, kidney) of five groups of mice were stained with H&E for investigating the biocompatibility of GA and ADSCs. All the slices with clot were counted as N. Slicings were scanning with a Leica DM6 microscope and volumes were measured with ImageJ software by two blinded investigators.

### Statistical analysis

Data were expressed as means ± SD and analyzed with GraphPad Prism (version 9.5.0, California, USA). Intergroup comparison was subject to one-way analysis of variance followed by Tukey post-hoc tests. Statistical significance was set at *P* < 0.05. All quantitative analyses were conducted by two independent investigators blinded to treatment groups.

## Results

### GA reduced MPP + induced cell injury and verification of ADSCs

**Fig. 1:**
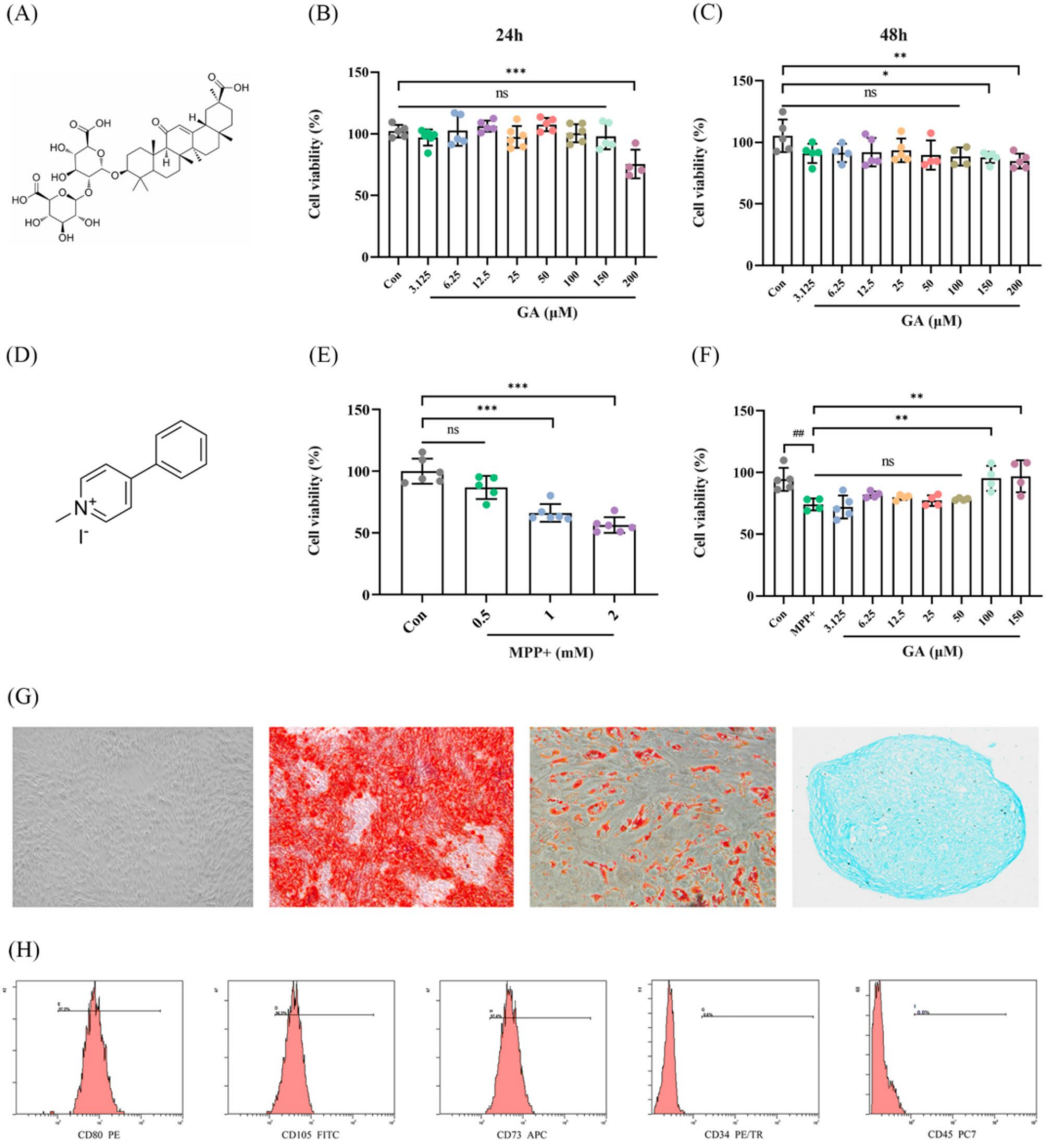
GA reduced MPP + induced cell injury in SY5Y cells. **A** Chemical structure of GA. **B** Cell viability after 24 h of GA treatment was measured by CCK-8 assay. *n* = 4, 5 or 6. **C** Cell viability after 48 h of GA treatment was measured by CCK-8 assay. *n* = 4 or 5. **D** Chemical structure of MPP+. **E** Cell viability after 24 h of MPP + treatment was measured by CCK-8 assay. *n* = 5 or 6. **F** Cell viability of MPP + cell model after GA treatment was measured by CCK-8 assay. *n* = 4 or 5. **G** Results of Oil red O, Alizarin Red, and Alcian Blue detection in cell cultures growing after 3 weeks. Control cells grew in regular medium. **H** The surface antigens of ADSCs. ^*^p < 0.05, ^**^p < 0.01 and ^***^p < 0.001 vs. Con; ^##^*p* < 0.01 and ^**^p < 0.01 vs. MPP+. Data are expressed as mean ± SD

The molecular structural formula of glycyrrhizic acid (GA) and MPP + iodide were shown in the Fig. 1A and D. Various concentrations (3.125, 6.25, 12.5, 25, 50, 100, 150µM) of GA did not show any cytotoxicity towards the neuroblastoma cell line SH-SY5Y after 24 h when compared to the control group. Conversely, GA at 200 µM could contribute to cytotoxicity in these cells (Fig. 1B). Additionally, various concentrations (3.125, 6.25, 12.5, 25, 50, 100 µM) of GA did not show any cytotoxicity towards SY5Y cells after 48 h when compared to the control group. Reversely, GA at 150 and 200 µM could contribute to cytotoxicity in SY5Y cells (Fig. 1C). Moreover, CCK-8 assay demonstrated that MPP + attenuated cell viability and increased cell death at a concentration greater than or equal to 1mM (Fig. 1E). To determine the effective concentration of GA for neuroprotection, SH-SY5Y cells were pretreated with 1 mM MPP + for 24 h and then exposed to GA at 3.125, 6.25, 12.5, 25, 50, 100, 150 µM for 24 h. We found that 100 and 150 µM of GA significantly ameliorated MPP+-induced neuronal cell death as compared with the MPP + exposure group (Fig. 1F). Therefore, the dosage of 100 µM GA was used in the following studies because of the best protective effect on MPP + damaged SY5Y cells. These results suggested that GA alleviated MPP+-induced cell injury of SY5Y cells. The differentiation potential of the ADSCs toward adipocytes, osteoblasts, and chondrocytes were demonstrated using corresponding induction media for 21 days. (Fig. 1G). Flow cytometry analyses revealed that the ADSCs were highly positive for CD73, CD105, CD90 and negative for CD45 and CD34 (Fig. 1H). The cells were verified as ADSCs.

### Combined GA and ADSCs alleviated neurotoxicity induced by MPP + in vitro

**Fig. 2:**
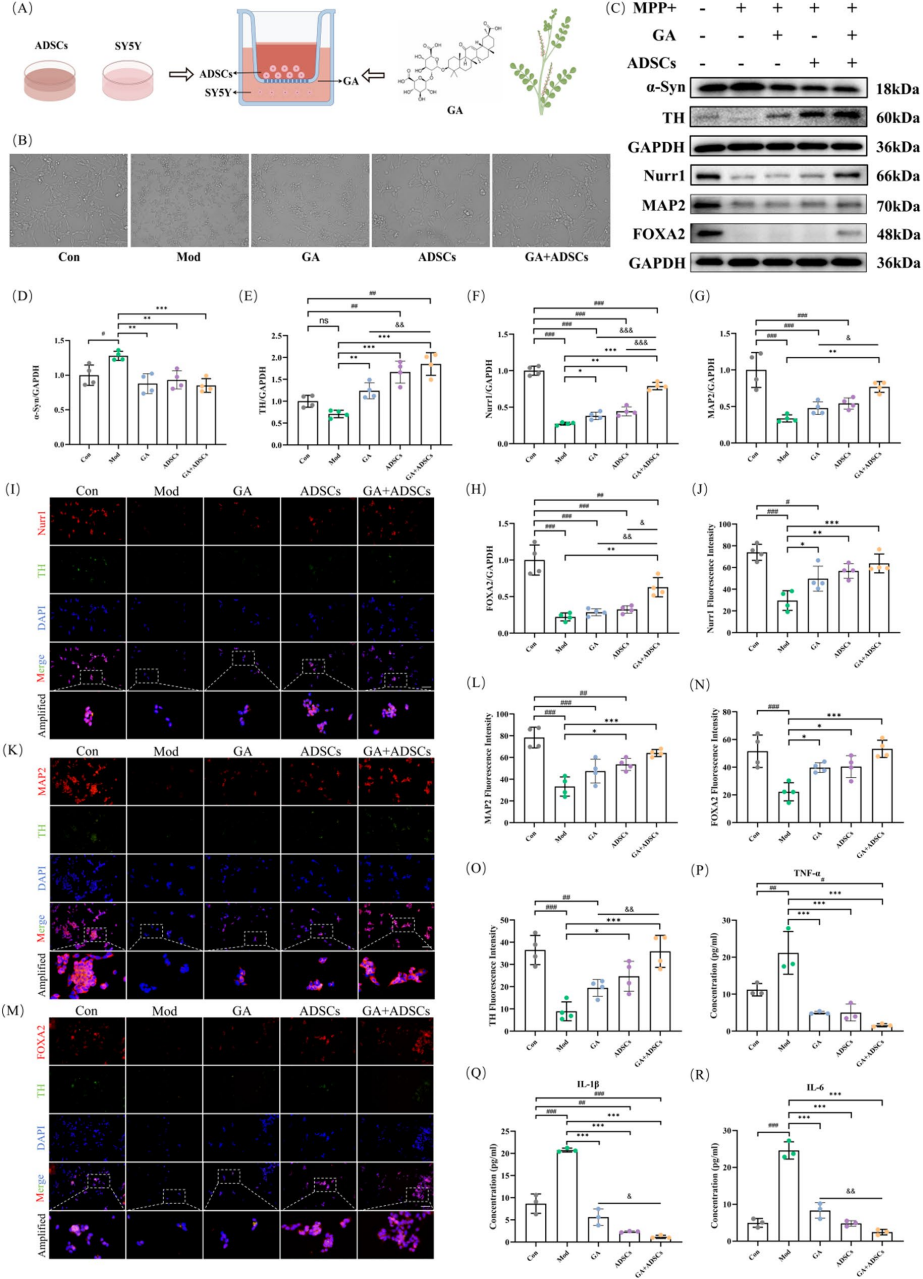
Combined GA and ADSCs alleviated neurotoxicity induced by MPP + in vitro. **A** Chematic diagram of the combined treatment of GA and ADSCs for SY5Y cells by Figdraw. Reprinted from Figdraw (www.figdraw.com) under a CC BY license, with permission from Figdraw, original copyright 2024. **B** Cell state under a imaging system (×100). **C** Representative expression of α-Syn, TH, Nurr1, MAP2 and FOXA2 protein detected by western blot. **D**-**H** Quantitative analysis of α-Syn, TH, Nurr1, MAP2 and FOXA2 protein expression. *n* = 4. **I**-**O** Representative expression of TH co-expressing with Nurr1, MAP2 and FOXA2 detected by immunofluorescence staining. *n* = 4 (Scale bar, 100 μm). **P**, **Q** and **R** Representative expression of TNF-α, IL-1β, and IL-6 detected by ELISA. *n* = 3. ^#^*p* < 0.05, ^##^*p* < 0.01 and ^###^*p* < 0.001 vs. Con; ^*^p < 0.05, ^**^p < 0.01 and ^***^p < 0.001 vs. Mod; ^&^*p* < 0.05, ^&&^*p* < 0.01 and ^&&&^*p* < 0.001 vs. GA + ADSCs. Data are expressed as mean ± SD

A schematic diagram of the combined GA and ADSCs treatment for SY5Y cells was shown in the Fig. 2A. ADSCs, GA and SY5Y cells were cocultured in vitro by transwell system for 24 h. Cell states under different treatments were detected by a imaging system (Fig. 2B). All treatment groups demonstrated significant reversal of the MPP+-induced neurodegenerative phenotype, which was characterized by extensive cell shrinkage, pronounced neurite retraction, and marked cellular clustering. Notably, the GA + ADSCs combination therapy showed superior restorative effects, achieving near-complete morphological recovery that closely resembled untreated control cells. These findings suggest GA and ADSCs act through complementary mechanisms to preserve neuronal integrity, with their combination providing optimal protection against MPP+-induced damage. Previous studies have reported that α-synuclein (α-Syn) plays a significant role in the pathology of Parkinson’s disease, while tyrosine hydroxylase (TH) is a well-established marker of dopaminergic neuronal integrity and function [38]. MAP2, a microtubule-associated protein, reflects neuronal integrity and is often diminished in PD models, indicating dendritic degeneration. NURR1 (NR4A2), a transcription factor critical for dopaminergic neuron development and maintenance, is frequently downregulated in PD, correlating with nigrostriatal dysfunction. FOXA2 and LMX1A, essential for midbrain dopaminergic differentiation and survival, exhibit altered expression in PD, suggesting their roles in disease progression and potential as therapeutic targets [39]. Therefore, we utilized western blot analysis to assess the expression of these proteins post-modeling (Fig. 2C). Full-length blots/gels are presented in Supplementary Figure S2. Our results showed a significant increase in α-Syn expression and marked decreases in TH, Nurr1, MAP2 and FOXA2 expression (Fig. 2D-H). In alignment with western blot results, IF test demonstrated a significant downregulation of TH co-expressing with Nurr1, MAP2 and FOXA2 (Fig. 2I-O). Furthermore, PCR test demonstrated a significant upregulation of *SNCA* and *LRRK2* and a downregulation of *TH*, *NURR1*, *MAP2*, *FOXA2*, *LMX1A*, *DAT* (Fig. S1D-K). Additionally, both GA and ADSCs treatments, administered either individually or in combination, reversed this trend. Interestingly, when addressing the neurotoxicity induced by MPP + modeling, the combined application of the two treatment strategies demonstrated significantly enhanced neuroprotective effects compared to the administration of either treatment alone. These results suggested that combined GA and ADSCs treatments were more effective in attenuating neurotoxicity induced by MPP + in vitro than GA and ADSCs alone. To further understand the mechanisms underlying the treatment combining GA and ADSCs, we used ELISA and PCR to observe the inflammation changes of the supernatant of SY5Y cells. Herein, many kinds of cytokines in the supernatant of SH-SY5Y cells were detected by ELISA (Fig. 2P-R) and PCR (Fig. S1L-N). Levels of TNF-α, IL-1β, and IL-6 were significantly upregulated in the model group compared with the control group (all *p* < 0.05). Treatment with GA, ADSCs, or their combination successfully reversed these cytokines expression changes. Notably, the co-administration of the GA and ADSCs resulted in superior anti-inflammatory effect against MPP+-induced neuroinflammation, outperforming the effects observed with either treatment used individually.

### Autophagy and PI3K/AKT/HIF-1α signaling pathway were involved in the improvement of SY5Y cells after the synergistic treatment of GA and ADSCs

**Fig. 3:**
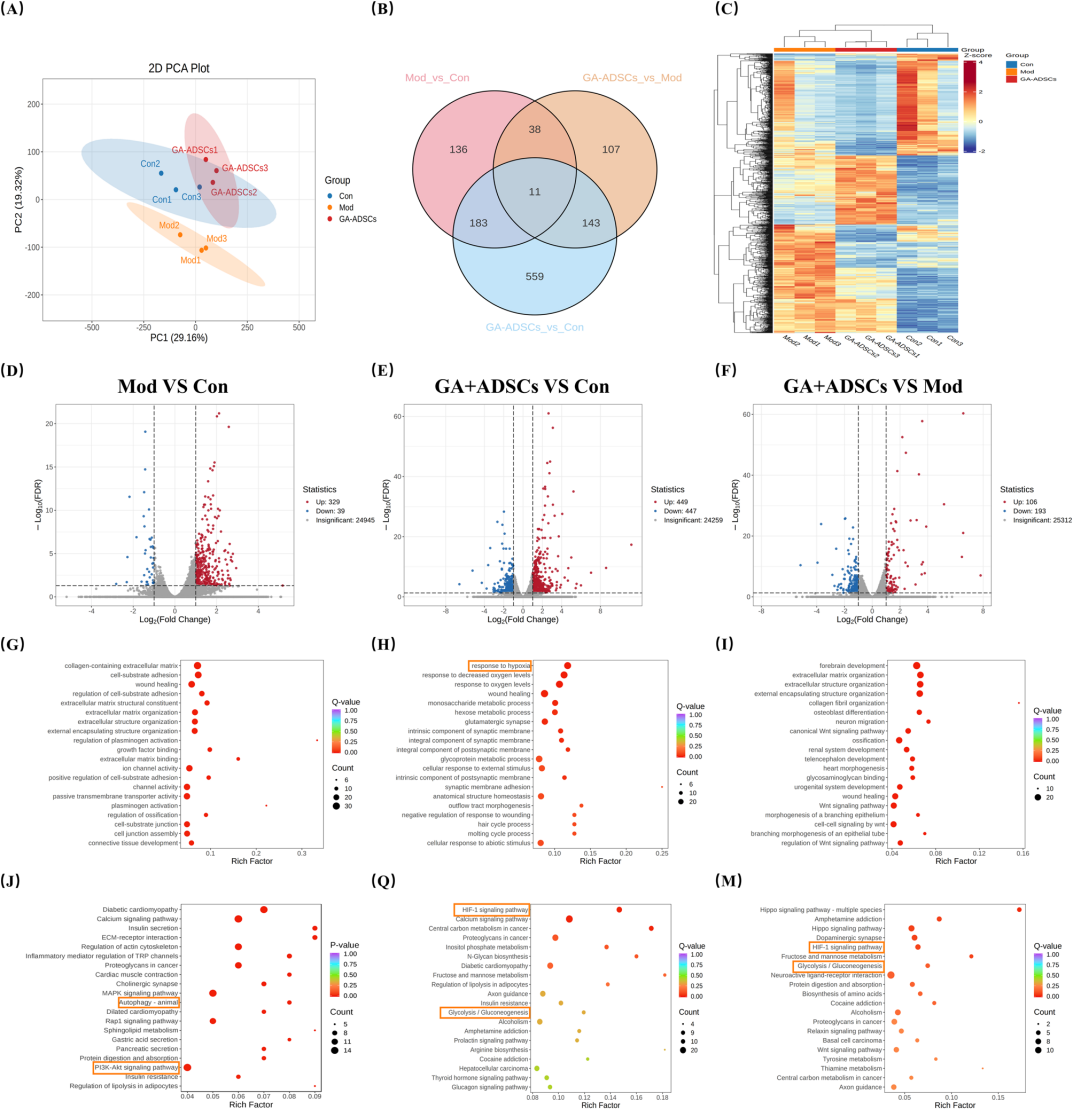
RNA-seq analysis for SH-SY5Y cells after combining GA and ADSCs treatments. **A** Principal component analysis. **B** Venn diagram. **C** Heatmap. **D**, **E** and **F** Volcano plot. **G**, **H** and **I** Gene ontology analysis. **J**, **Q** and **M** Gene ontology analysis

The molecular mechanisms of neurotoxicity induced by MPP + alleviation by using mixed GA and ADSCs treatment were evaluated. We identified DEGs in the treated SH-SY5Y cell samples through RNA sequencing and analysis (Fig. 3). Principal component analysis (PCA) of the gene expression profile showed significant separations among the Con, Mod and GA + ADSCs groups. Compared with the Mod group, the GA + ADSCs cluster was closer to the Con group, indicating that the gene expression of GA + ADSCs group was more similar to the Con group (Fig. 3A). To identify shared and unique gene expression profiles among the three experimental groups (Mod VS Con, GA + ADSCs VS Con, and GA + ADSCs VS Mod), a Venn diagram was generated. A total of 11 genes were found to be commonly expressed across all groups, while 136 genes were uniquely expressed in the Mod VS Con group, 559 genes in the GA + ADSCs VS Con group, and 107 genes in the GA + ADSCs VS Mod group (Fig. 3B). A total of 1,177 DEGs were identified for further analysis in the SY5Y cells. A heatmap was generated to visualize the expression patterns of the differentially expressed genes (DEGs) among the Con, Mod, and GA + ADSCs groups. Hierarchical clustering showed that the Mod group exhibited distinct gene expression changes compared to the Con, with significant upregulation and downregulation of specific genes. The GA + ADSCs group, however, demonstrated a gene expression profile more similar to the Con group, suggesting that GA + ADSCs treatment partially reversed the Mod-induced changes (Fig. 3C). A total of 39 genes were down-regulated and 329 up-regulated in the Mod group compared to the Con group, while 447 genes were down-regulated and 449 up-regulated in the GA + ADSCs group compared to the Con group. Compared to the Mod group, the GA + ADSCs group exhibited 193 down-regulated and 106 up-regulated genes, indicating substantial differences in therapeutic mechanisms (Fig. 3D-F). Gene Ontology (GO) enrichment analysis was performed on the differentially expressed genes to identify significantly enriched biological processes, molecular functions, and cellular components. GO enrichment analysis revealed collagen-containing extracellular matrix, response to hypoxia and Wnt signaling pathway in the up-regulated and down-regulated DEGs (Fig. 3G–I). KEGG pathway enrichment analysis indicated that Autophagy, PI3K/AKT signaling pathway, HlF-1 signaling pathway and Glycolysis/Gluconeogenesis enriched in the up-regulated and down-regulated DEGs (Fig. 3J-M). The results in vitro further showed that autophagy and PI3K/AKT/HIF-1α signaling pathway may were involved in the neurotoxicity induced by MPP + in SY5Y cells in treatments combining GA + ADSCs.

### Combined treatment with GA and ADSCs regulated PI3K/AKT/HIF-1α pathway and relieved autophagy in SY5Y cells

**Fig. 4:**
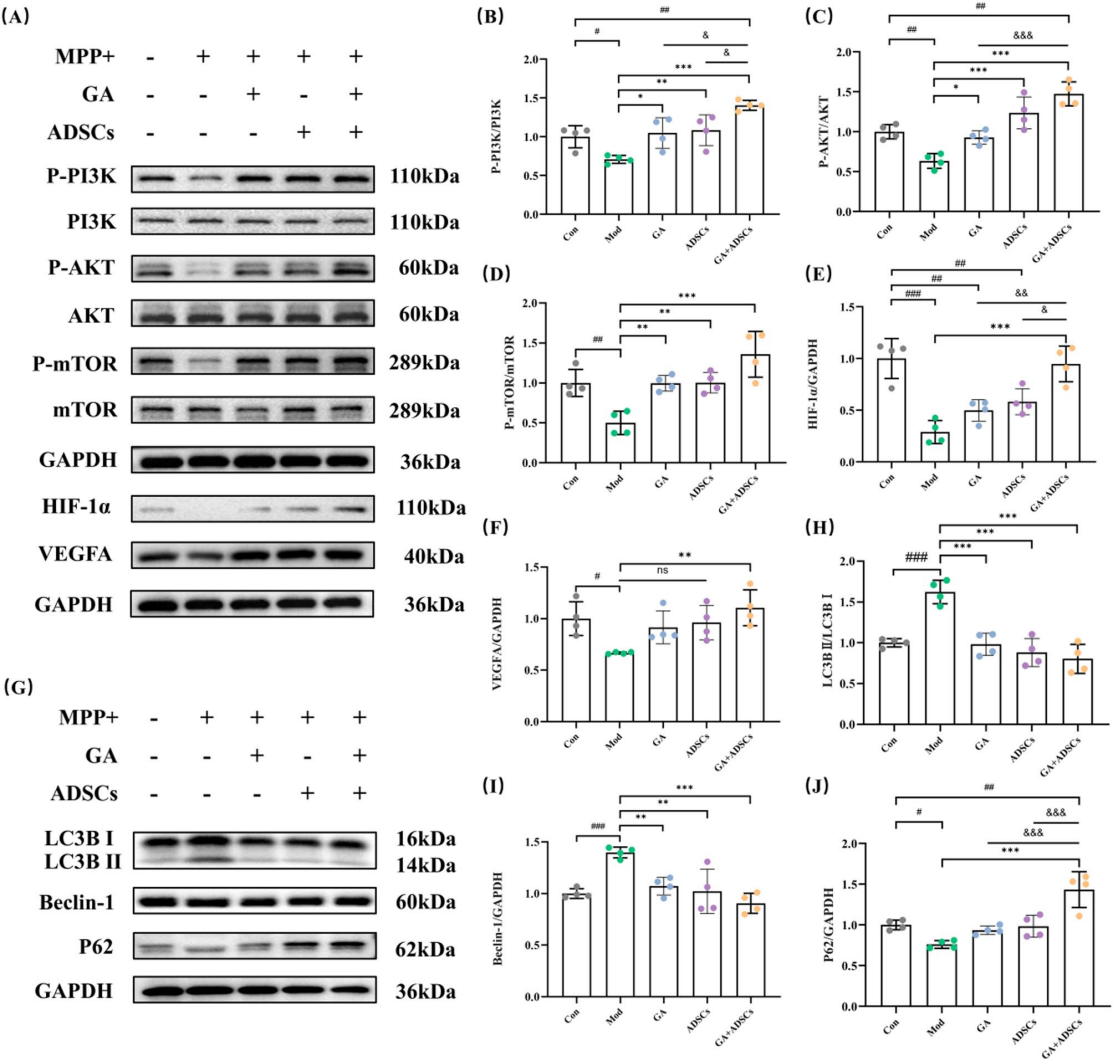
Combined treatment with GA and ADSCs regulated PI3K/AKT/HIF-1α pathway and relieved autophagy in SY5Y cells. **A** Representative expression of p-PI3K, PI3K, p-AKT, AKT, p-mTOR, mTOR, HIF-1α and VEGFA detected by western blot. **B**–**F** Statistics on the relative expression of p-PI3K, p-AKT, p-mTOR, HIF-1α and VEGFA. *n* = 4. **G** Representative expression of LC3B II/I, Beclin-1 and P62 detected by western blot. **H**, **I** and **J** Statistics on the relative expression of LC3B II/I, Beclin-1 and P62. *n* = 4. ^#^*p* < 0.05, ^##^*p* < 0.01 and ^###^*p* < 0.001 vs. Con; ^*^p < 0.05, ^**^p < 0.01 and ^***^p < 0.001 vs. Mod; ^&^*p* < 0.05, ^&&^*p* < 0.01 and ^&&&^*p* < 0.001 vs. GA + ADSCs. Data are expressed as mean ± SD

Subsequently, the molecular mechanisms by which GA and ADSCs operated in SY5Y cells induced by MPP + were examined (Fig. 4). Western blot analysis demonstrated that p-PI3K/PI3K, p-AKT/AKT, p-mTOR/mTOR expression ratio and HIF-1α, VEGFA expression level all decreased following MPP+. The phosphorylation site of PI3K, AKT and mTOR is Tyr458, Ser473, and Ser2448, respectively. Either GA or ADSCs, or a combination of both, all alleviated the decline in phosphorylation levels. Interestingly, in comparison to GA or ADSCs monotherapy, the combined administration of GA and ADSCs exhibited significantly superior improvement in phosphorylation levels (Fig. 4A–F). Full-length blots/gels are presented in Supplementary Figure S2. The subsequent PCR experiment results further validated the findings of the previous experiments, showing consistency with the results from the Western blot analysis (S1 Fig). Moreover, autophagy activity was considerably enhanced by MPP+, including up-regulation of the ratio of LC3B II to I and the level of Beclin-1, down-regulation of the P62 protein level. Compared to their model counterparts, GA, ADSCs or both treatment discernibly reversed the up-regulation of the ratio of LC3B II to I and the level of Beclin-1and einstated the diminished level of P62 (Fig. 4G–J). The combined use of GA and ADSCs demonstrates a more pronounced improvement in mitigating these effects. Therefore, the protective effects and mechanisms of GA and ADSCs in MPP+-induced damage may have participated in the autophagy and PI3K/AKT/HIF-1α pathway.

### Combined treatment with GA and ADSCs suppressed autophagy in SH-SY5Y cells

**Fig. 5:**
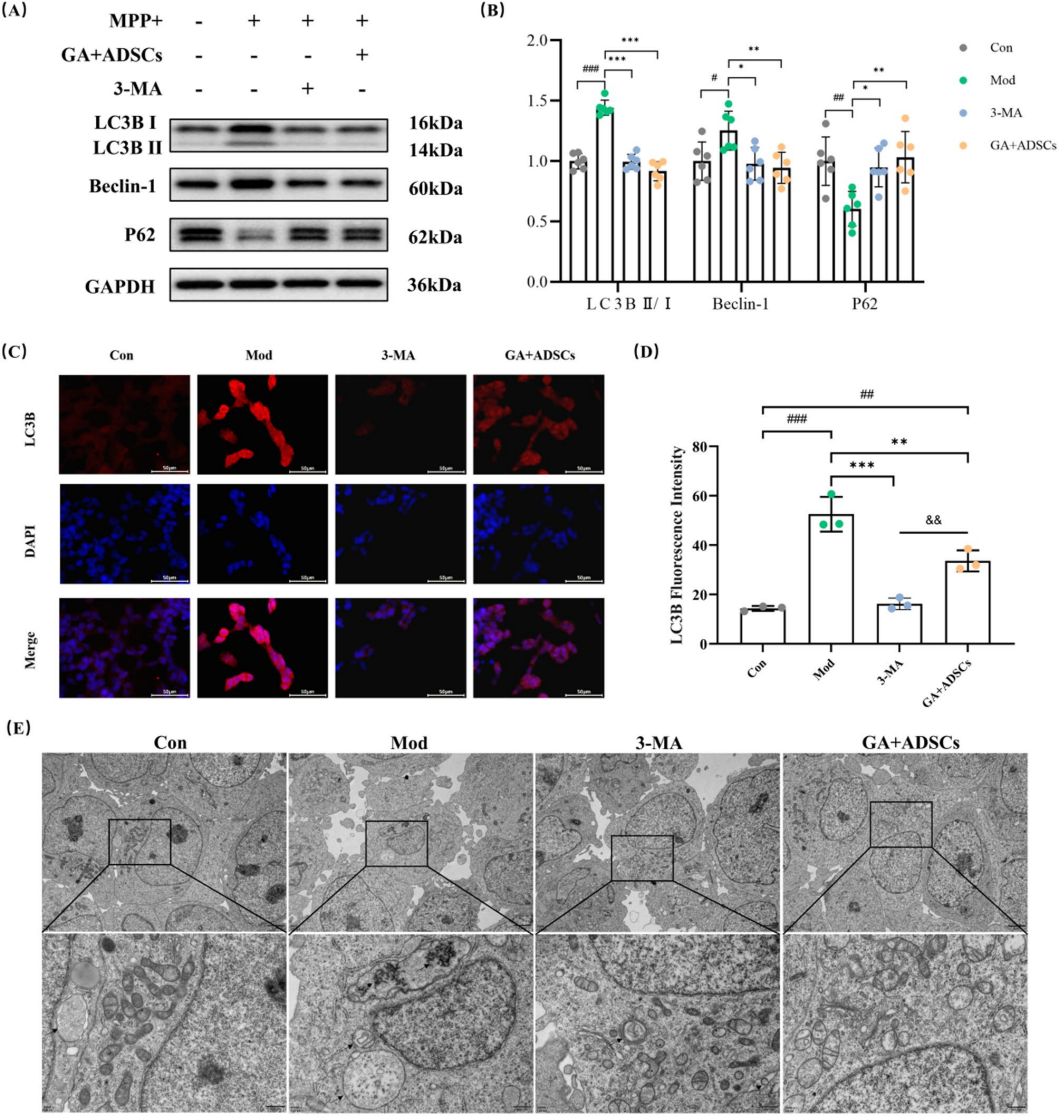
Combined treatment with GA and ADSCs suppresses autophagy in SH-SY5Y cells. **A** Representative expression of LC3B II/I, Beclin-1 and P62 detected by western blot. **B** Statistics on the relative expression of LC3B II/I, Beclin-1 and P62. *n* = 6. **C** Cell immunofluorescence for LC3B (Scale bar, 50 μm). **D** Statistics on the relative fluorescence intensity of LC3B. *n* = 3. **E** Ultrastructural features captured by TEM in SH-SY5Y cells (the black arrow indicates autophagosomes and autolysosomes, Scale bar, 2 μm or 500 nm). ^#^*p* < 0.05, ^##^*p* < 0.01 and ^###^*p* < 0.001 vs. Con; ^*^p < 0.05, ^**^p < 0.01 and ^***^p < 0.001 vs. Mod; ^&&^*p* < 0.01 vs. GA + ADSCs. Data are expressed as mean ± SD

Previous studies have reported that autophagy might be a potential therapeutic target for parkinson’ s disease. 3-MA, widely recognized as an autophagy blocking agent, has been extensively utilized in numerous studies focusing on autophagy mechanisms. Therefore, we delved into the investigation of the role of autophagy in mediating neuroprotective effect by combined GA and ADSCs. Moreover, autophagy activity was considerably enhanced by MPP+, including increased percentage of the cells with LC3B puncta (Fig. 5C and D), up-regulation of the level of Beclin-1 and ratio of LC3B II to I, down-regulation of the P62 protein (Fig. 5A and B). Full-length blots/gels are presented in Supplementary Figure S2. Following MPP + damage, SY5Y cells produced additional autophagosomes and autolysosomes, according to electron microscopic analysis (Fig. 5E). In contrast, both GA + ADSCs and 3-MA remarkably reduced the number of autophagosome and autolysosome. Consistent with the results of TEM, GA + ADSCs and 3-MA administration decreased the expression ratio of LC3B II/I and the level of Beclin-1, and activated P62 level (Fig. 5A and B). Hence, GA + ADSCs generates a comparable effect to that of 3-MA. These findings further demonstrated that GA + ADSCs protected MPP+-injured SY5Y cells by inhibiting autophagy.

### Combined treatment with GA and ADSCs protected against MPP+-induced SY5Y cells injury by activation of the PI3K/AKT/HIF-1α pathway

**Fig. 6:**
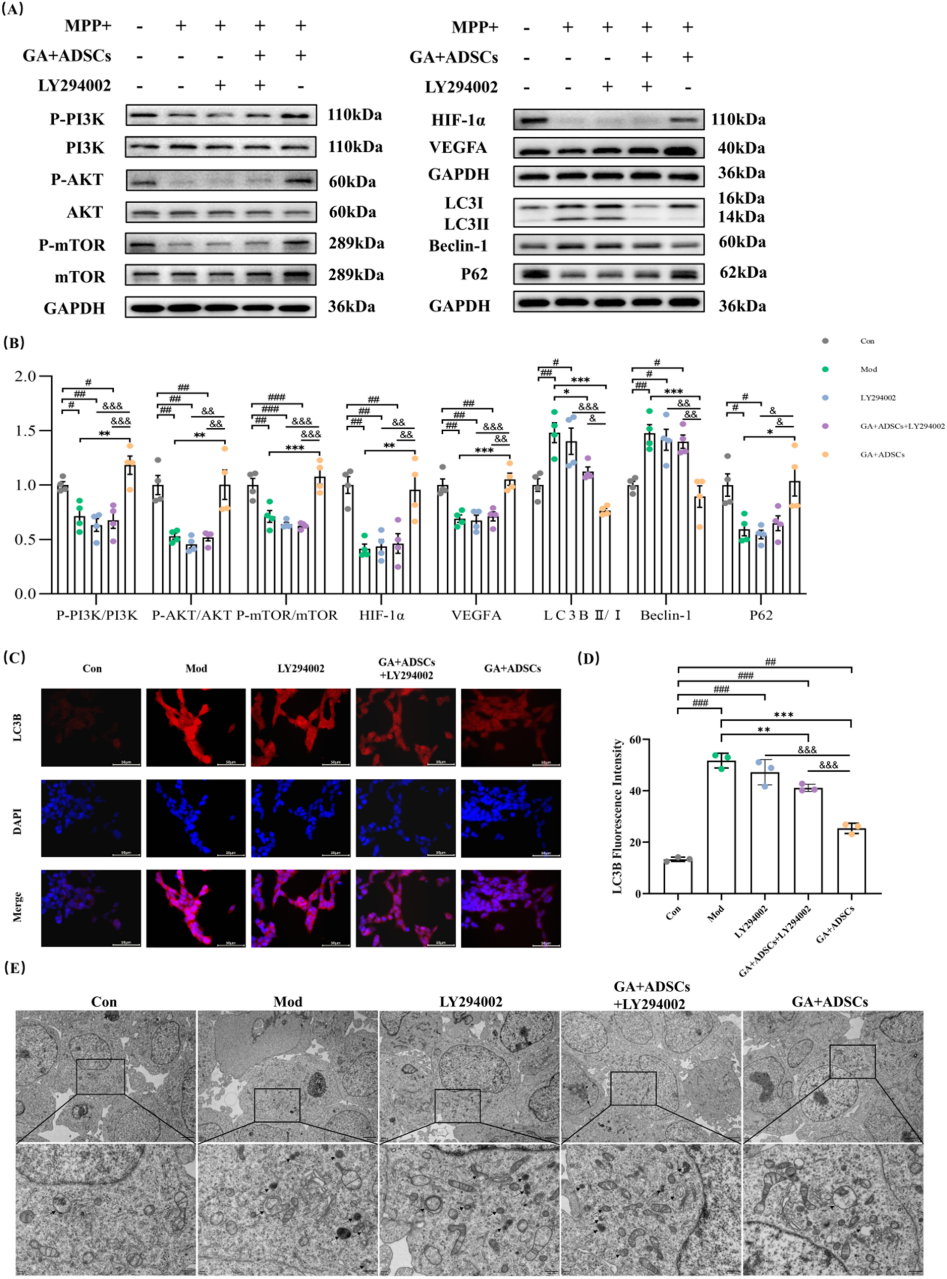
Combined treatment with GA and ADSCs protected against MPP+-induced SY5Y cells injury by activation of the PI3K/AKT/HIF-1α pathway. **A** Representative expression of p-PI3K, PI3K, p-AKT, AKT, p-mTOR, mTOR, HIF-1α, VEGFA, LC3B II/I, Beclin-1 and P62 detected by western blot. **B** Statistics on the relative expression of p-PI3K, p-AKT, p-mTOR, HIF-1α, VEGFA, LC3B II/I, Beclin-1 and P62. *n* = 4. **C** Cell immunofluorescence for LC3B (Scale bar, 50 μm). **D** Statistics on the relative fluorescence intensity of LC3B. *n* = 3. **E** Ultrastructural features captured by TEM in SH-SY5Y cells (the black arrow indicates autophagosomes and autolysosomes, Scale bar, 2 μm or 500 nm). ^#^*p* < 0.05, ^##^*p* < 0.01 and ^###^*p* < 0.001 vs. Con; ^*^p < 0.05, ^**^p < 0.01 and ^***^p < 0.001 vs. Mod; ^&^*p* < 0.05, ^&&^*p* < 0.01 and ^&&&^*p* < 0.001 vs. GA + ADSCs. Data are expressed as mean ± SD

As a PI3K-specific inhibitor, LY294002 effectively obstructs the phosphorylation activation of PI3K targets and concurrently inhibits the activation of downstream signaling pathways, including the PI3K/AKT/HIF-1α pathway. Hence, our findings elucidated that LY294002 effectively obstructed the phosphorylation activation of PI3K/AKT/HIF-1α pathway (Fig. 6A and B). Full-length blots/gels are presented in Supplementary Figure S2. To further investigate the impact of PI3K activation on alleviating autophagy under GA + ADSCs treatment in vitro, we observed that both the model and LY294002 groups exhibited a significant increase in LC3B-positive SY5Y cells (Fig. 6C and D). In contrast, GA + ADSCs treatment notably reversed this effect after MPP + exposure (Fig. 6A and B). Consistent with the immunofluorescence results, LY294002 partially reversed the down-regulation of the LC3B II/I ratio, Beclin-1 levels, and the up-regulation of p62 in SY5Y cells treated with GA + ADSCs following MPP + exposure (Fig. 6A and B). Correspondingly, LY294002 significantly reversed the down-regulation of additional autophagosomes and autolysosomes induced by GA + ADSCs treatment (Fig. 6E). These findings suggest that GA + ADSCs may inhibit autophagy in MPP+-induced SY5Y cells via the PI3K/AKT/HIF-1α signaling pathway.

### Combined treatment with GA and ADSCs attenuated PD-like behavioral disorder in MPTP model mice

**Fig. 7:**
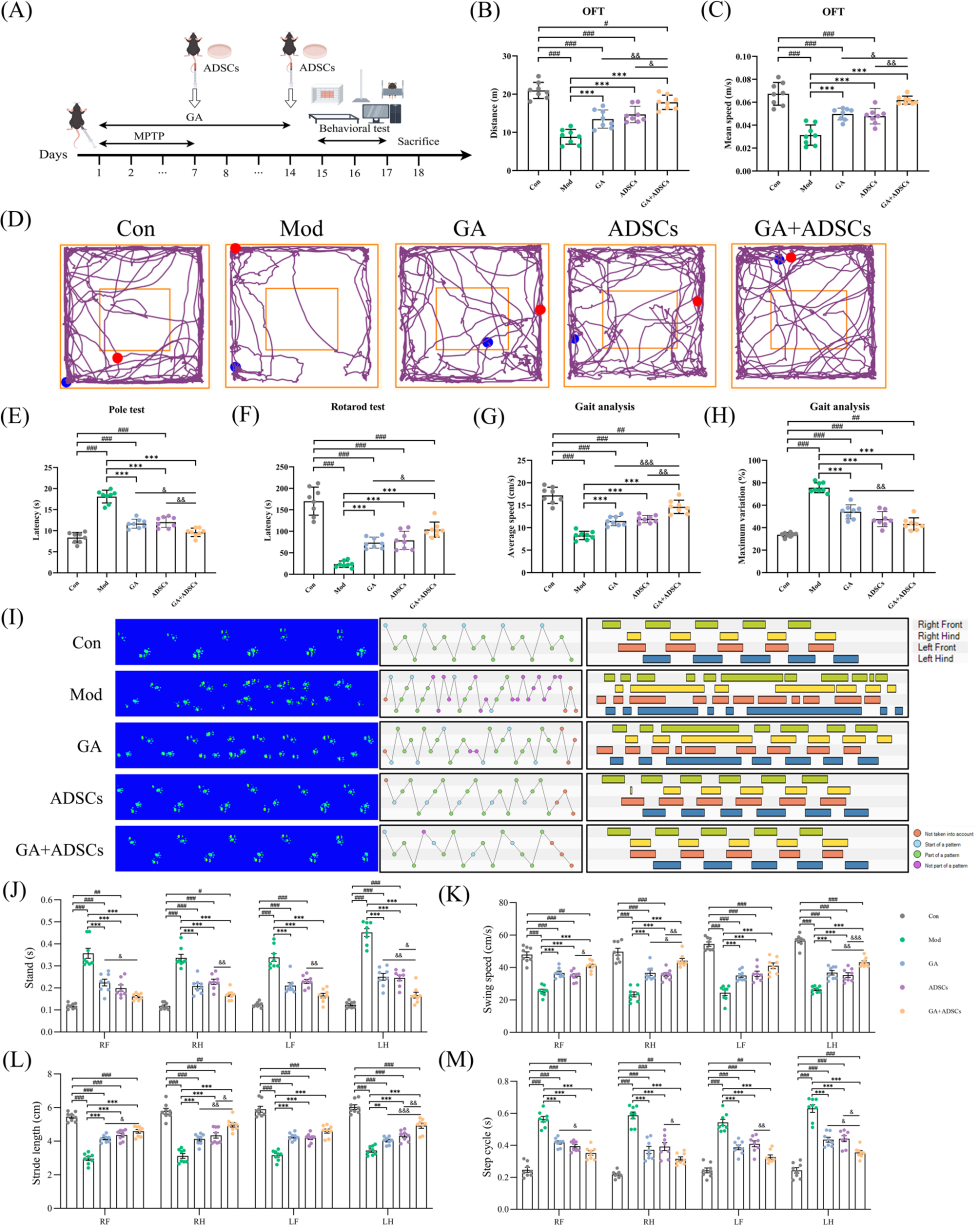
Combined treatment with GA and ADSCs attenuated PD-like behavioral disorder in MPTP model mice. **A** Time course of the experimental procedure by Figdraw. Reprinted from Figdraw (www.figdraw.com) under a CC BY license, with permission from Figdraw, original copyright 2024. Mice were initially treated with MPTP for 7 days, followed by treatment with GA, ADSCs or both. **B** The total distance traveled for 5 min of the indicated mice in the open field test. *n* = 8. **C** The mean speed of the indicated mice in the open field test. *n* = 8. **D** Representative track images of the indicated mice in open field test. **E** Periods needed for the mice to descend from the pole were documented in the pole test. *n* = 8. **F** Durations needed for the mice to walk on the cylindrical rotating rotarod were documented in the rotarod test. *n* = 8. **G** Average speed was assessed by gait analysis. *n* = 8. **H** Maximum variation was assessed by gait analysis. *n* = 8. **I** The gait pattern was visualized as footprint view and footprint pattern. RF, right front. RH, right hind. LF, left front. LH, left hind. Green balls: part of a pattern. Red balls: abnormal pattern. Blue balls: start of a pattern. Purple balls: not part of a pattern. **J**-**M** Disordered motor coordination of mice was assessed by gait analysis including the stand (**J**), swing speed (**K**), stride length (**L**) and step cycle (**M**). *n* = 8. ^#^*p* < 0.05, ^##^*p* < 0.01 and ^###^*p* < 0.001 vs. Con; ^***^p < 0.001 vs. Mod; ^&^*p* < 0.05, ^&&^*p* < 0.01 and ^&&&^*p* < 0.001 vs. GA + ADSCs. Data are expressed as mean ± SD

To experimentally confirm the functional impacts of the combined treatment with GA and ADSCs on PD in vivo, we employed a widely-used neurotoxin-based PD mice model induced by MPTP, to rapidly induce dopaminergic neuron damage (Fig. 7A). This model is particularly useful for the evaluation of drug efficacy in motor function restoration. Behavioral tests, including the open field test, pole test, rotarod test, and gait analysis, confirmed typical motor impairments in MPTP model (Fig. 7B-M). In PD model mice, both GA and ADSCs significantly improved motor function and alleviated gait disturbances compared to the untreated group. Specifically, the combined treatment with GA and ADSCs restored motor function to levels comparable to single employment of GA or ADSCs, as indicated by a quantitative improvement in distances and mean speeds in arena, pole descent times, increased endurance on the rotarod, and enhanced gait parameters (Fig. 7B-M). Together, these data strongly indicated that the combined treatment with GA and ADSCs play a beneficial role in alleviating parkinsonism in PD model mice.

### GA and ADSCs treatment attenuated dopaminergic neuronal degeneration

**Fig. 8:**
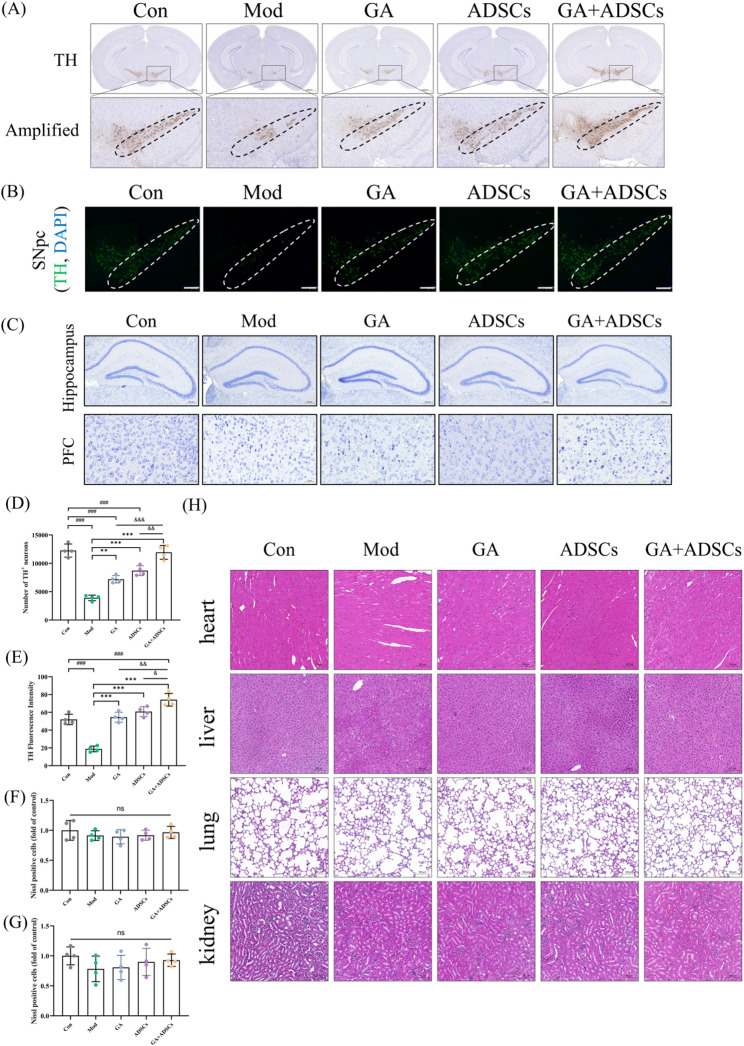
GA and ADSCs treatment attenuated dopaminergic neuronal degeneration. **A** Immunohistochemistry analysis of TH expression in the SNpc. Representative images were shown. Scale bar, 1000 μm. *n* = 4. **B** Immunostaining analysis of TH expression (green) in the SNpc. DAPI (blue) was used to identify the nucleus. Scale bar, 200 μm. *n* = 4. **C** Representative images of Nissl staining of subregions of the hippocampus and prefrontal cortex (PFC). Scale bar, 200 and 50 μm. *n* = 4. **D** Quantitative analysis of TH-positive neurons in the SNpc. **E** Quantification of relative TH intensity in the SNpc. **F** and **G** Quantitative analysis of the number of Nissl + cells in the hippocampus and cortex. **H** The histopathology changes in the heart, liver, lung, and kidney of mice were detected using H&E staining. Scale bars: 100 μm. ^###^*p* < 0.001 vs. Con; ^**^p < 0.01 and ^***^p < 0.001 vs. Mod; ^&^*p* < 0.05, ^&&^*p* < 0.01 and ^&&&^*p* < 0.001 vs. GA + ADSCs. Data are expressed as mean ± SD

**Fig. 9:**
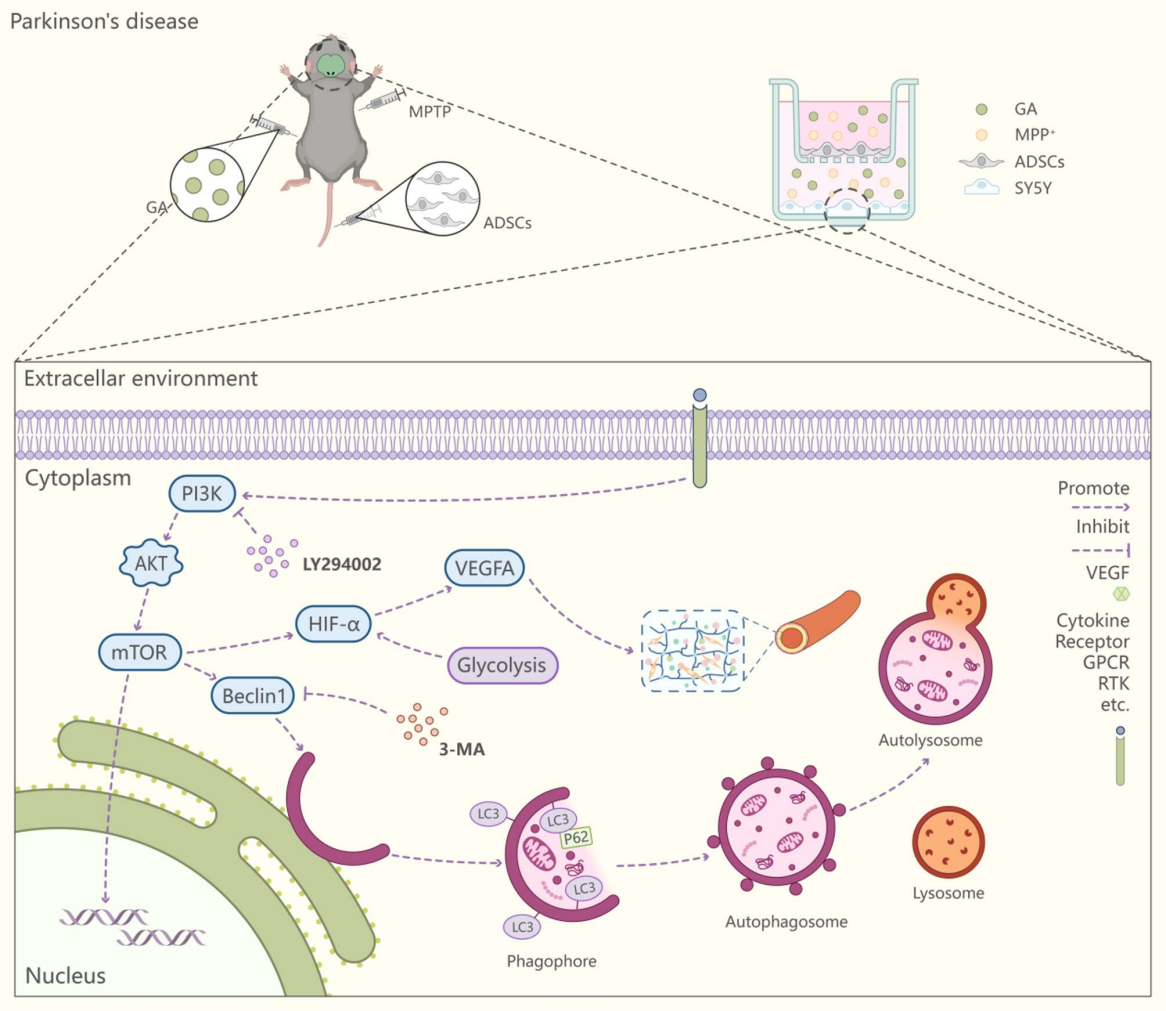
Schematic illustration of the potential protective effects of GA and ADSCs in Parkinson’s disease

Degenerating neurons are the primary cause of dopamine deficiency in the nigrostriatal pathway, which leads to motor dysfunction. To evaluate the neuroprotective effects of GA and ADSCs intervention, we quantified the number of dopaminergic neurons in the midbrain, using both immunofluorescence and immunohistochemistry to measure tyrosine hydroxylase (TH). In MPTP-induced PD model mice, tyrosine hydroxylase (TH) expression was decreased in the SNpc. However, in the presence of GA and ADSCs, this reduction was substantially prevented. It is worth noting that the combined use of GA and ADSCs demonstrated more pronounced effects (Fig. 8A, B, D and E). In addition, our data showed after 2 weeks of GA treatment or 2 rounds of ADSCs treatment in the MPTP model, no significant histopathological changes in the hippocampus, prefrontal cortex, heart, liver, lung, and kidney of mice were observed, indicating no apparent toxicity to organs within the dosing and duration used in this study (Fig. 8C, F, G and H). Together, these data observably indicated that the combined treatment with GA and ADSCs attenuated dopaminergic neuronal degeneration in PD model mice (Fig. 9).

## Discussion

Glycyrrhizic acid (GA), a compound derived from the traditional Chinese medicinal herb *Glycyrrhiza glabra*, which has been widely utilized in Asia, where it is a key component of numerous herbal formulations. GA and ADSCs have demonstrated strong anti-inflammatory and antiviral properties, making them effective in treating inflammatory conditions and neurodegenerative diseases [40, 41]. However, the exact role of GA and ADSCs in the treatment of PD, including their mechanisms and therapeutic pathways, remains unclear. This study is the first to explore the combined application of GA and ADSCs in a MPP+/MPTP-induced neurotoxic PD model in vitro and in vivo. Notably, our findings suggested that the combination of GA and ADSCs could suppress MPP+-induced excessive autophagy by modulating the PI3K/AKT/HIF-1α signaling pathway.

The SH-SY5Y cell line, derived from a neuroblastoma patient, exhibits limited neuronal differentiation potential, and many studies utilize these cells in their undifferentiated state—raising concerns about their validity as neuronal models [42]. Furthermore, the oncogenic background of SH-SY5Y cells introduces genetic aberrations that may influence differentiation, viability, and metabolic function, complicating their translational relevance. The emergence of human induced pluripotent stem cell (hiPSC)-based neuronal models now offers a genetically stable and donor-specific alternative, more accurately reflecting human neurobiology. Thus, while SH-SY5Y cells remain valuable for exploratory research, hiPSC-derived neurons provide a superior platform for translational studies [43].

Despite promising results, the application of MSCs in neurological diseases such as Parkinson’s disease remains controversial, largely due to inconsistent therapeutic outcomes, potential tumorigenicity, and translational limitations arising from cellular heterogeneity and lack of standardized protocols [44]. To overcome these challenges, researchers are increasingly exploring cell-free strategies by harnessing the secretory potential of MSCs through extracellular vesicles (EVs) and exosomes [45]. As a potential alternative, the use of MSC-derived extracellular vesicles (EVs) may help mitigate these concerns, offering a cell-free therapeutic strategy with reduced safety risks and improved reproducibility [46].

Autophagy is involved in various neurodegenerative diseases, including PD, Alzheimer’ s disease, and multiple system atrophy [47]. Increasing evidence points to its pivotal role in the pathogenesis of PD. However, the specific function and regulatory mechanisms of autophagy in PD remain controversial. Autophagy, widely known as a “double-edged sword,” functions as a protective system in response to mild stress by clearing abnormal cells. While autophagy serves protective functions, excessive activation may disrupt normal cellular processes, compromise neuronal function, and contribute to disease progression [48]. Studies have shown that the autophagy inhibitor 3-MA improves motor function in PD mouse models [49]. Our findings suggested that while excessive autophagy aggravated neuronal damage in PD, treatment with GA and ADSCs significantly reduced MPP+-induced neurotoxicity by inhibiting autophagy, supporting cell viability, and preserving normal cell phenotypes. This combined neuroprotective effect against MPP+-induced damage in SY5Y cells emphasizes the necessity for continued exploration of the underlying mechanisms.

As a highly conserved cellular degradation pathway, autophagy is controlled by a multitude of autophagy-associated proteins [50]. Beclin-1 serves as a primary regulator, initiating the formation of autophagosomes by interacting with the Vps34 complex. LC3 undergoes ATG4-mediated lipidation to become LC3-I, which is then converted into LC3-II within autophagosomes, marking autophagosome formation [51]. The polyubiquitin-binding protein p62 interacts with LC3, aiding in the degradation of ubiquitin aggregates during autophagy. The expression and activity of Beclin-1 are strongly associated with LC3-II levels, and dysregulation of Beclin-1 can lead to autophagic disorders [52]. As a polyubiquitin-binding protein, p62 binds to LC3, thereby promoting the selective degradation of ubiquitin aggregates within the autophagic process [53]. Our research showed that GA + ADSCs treatment reversed the MPP+-induced changes in the LC3-II/LC3-I ratio, as well as in the levels of p62 and Beclin-1. In addition, autophagosome numbers in MPP+-treated SH-SY5Y cells significantly decreased following 3-MA treatment, while GA + ADSCs similarly reduced both autophagosome and autolysosome formation, mirroring the effects of 3-MA. Building upon these findings, it seems that the inhibition of autophagy—especially through the modulation of the Beclin-1-dependent pathway—holds a pivotal role in the neuroprotective effects of GA + ADSCs against Parkinson’s disease. Although our data indicate that autophagy modulation is a crucial component, both GA and ADSCs exhibit multimodal neuroprotective effects, including ameliorating neuroinflammation, promoting trophic factor secretion, immunomodulation and mitochondrial function regulation. The superior synergistic efficacy of GA + ADSCs (compared to individual treatment) likely stems from the combination of GA’s direct autophagy inhibition with ADSCs’ paracrine support, potentially further enhanced by GA-mediated ADSCs priming through optimized miRNA profiles and increased exosome release. In contrast to 3-MA, our combined approach demonstrates greater clinical potential by overcoming 3-MA’s limitations of poor pharmacokinetics, non-specificity, and hepatotoxicity, while providing comprehensive neuroprotection through complementary pathways [54]. Future studies will focus on optimizing these autophagy-targeting strategies for scientific and clinical applications.

mTOR stands as a master conductor of autophagy, harmonizing inputs from diverse signaling pathways—most notably those that measure the energy pulse of cell—to regulate the symphony of protein creation [55]. Phosphorylation of mTOR modulates autophagy by altering the LC3II/I expression ratio. According to our results, MPP + decreased the p-mTOR/mTOR ratio, a reduction that GA + ADSCs treatment then countered [56]. Furthermore, mTOR functions as a downstream target of the PI3K/AKT pathway, which is activated by neurotrophic and growth factor receptors, facilitating cell growth, differentiation, and survival, while inhibiting apoptotic pathways. Therefore, activation of the PI3K/AKT/mTOR pathway theoretically promotes cell survival, neuroprotection, and autophagy inhibition through mTOR activation. Our study revealed that MPP + notably lowered p-PI3K, p-AKT, and p-mTOR levels, an effect that was reversed by GA + ADSCs treatment, implying that GA + ADSCs might confer neuroprotection via the PI3K/AKT/mTOR pathway. Nonetheless, the mTOR pathway integrates multiple regulatory signals and serves as a crucial switch for upstream pathways like PI3K/AKT, underscoring its importance in devising targeted therapeutic approaches.

Transcriptomic analysis indicated the involvement of HIF-1α and its downstream targets in the therapeutic effects. As a hypoxia-inducible transcription factor, HIF-1α plays a critical role in supporting cell survival and proliferation. It has been shown that HIF-1α and its downstream targets are downregulated in substantia nigra neurons among PD patients. Notably, HIF-1α accumulation was inhibited in dopaminergic PC12 cells and in mice in the MPTP-induced PD model. The interaction between HIF-1α and tyrosine hydroxylase (TH) forms a crucial bridge to understanding its role in Parkinson’s disease. However, it remains unclear whether GA + ADSCs affect autophagy via the PI3K/AKT/HIF-1α signaling pathway. To assess the causal relationship between GA + ADSCs and the PI3K/AKT/HIF-1α signaling pathway, we employed in vitro experiments with the PI3K-specific inhibitor LY294002. LY294002 reversed the positive effects of GA + ADSCs after MPP + injury. Our results showed that GA + ADSCs activated the PI3K/AKT/HIF-1α signaling pathway, which was inhibited by MPP + injury. This study provides new insights into PD treatment, especially for therapies combining Traditional Chinese Medicine with cell-based approaches.

## Conclusions

In conclusion, our findings present the first evidence of the neuroprotective effects of GA and ADSCs in alleviating PD in vitro and in vivo, potentially linked to autophagy suppression. Notably, we have demonstrated that the neuroprotective effects of GA and ADSCs might be related to the PI3K/AKT/HIF-1α signaling pathway. Through their pronounced neuroprotective effects, this combination demonstrates promising potential for PD therapy. Ultimately, this work not only holds great interest for researchers in neurodegenerative diseases but also offers significant implications for stem cell therapy and Traditional Chinese Medicine monomer studies.

## Supplementary Information

Supplementary material 1.

## Data Availability

The RNA-seq data supporting the findings of this study have been deposited in the NCBI Sequence Read Archive (SRA) under the BioProject accession number PRJNA1231711 (https://www.ncbi.nlm.nih.gov/bioproject/PRJNA1231711). All other data supporting the results of this study are included within the article and its supplementary materials.

## Abbreviations

GA: Glycyrrhizinic acid
MSCs: Mesenchymal stem cells
ADSCs: Adipose-derived mesenchymal stem cells
MPP+: 1-methyl-4-phenylpyridinium
MPTP: 1-methyl-4-phenyl-1,2,3,6-tetrahydropyridin
PD: Parkinson’s disease
3-MA: 3-methyladenine
CCK-8: Cell counting kit-8
mTOR: Mammalian target of rapamycin
PI3K: Phosphoinositide-3-kinase
SPF: Specified pathogen free
TEM: Transmission electron microscopy
qRT-PCR: Quantitative reverse transcription polymerase chain reaction
IF: Immunofluorescence Staining
CNS: Central nervous system

## References

[1] Ben-Shlomo Y, Darweesh S, Llibre-Guerra J, Marras C, San Luciano M, Tanner C. The epidemiology of Parkinson’s disease. Lancet. 2024;403(10423):283–92. 38245248 10.1016/S0140-6736(23)01419-8 PMC11123577

[2] Bhidayasiri R, Sringean J, Phumphid S, Anan C, Thanawattano C, Deoisres S, et al. The rise of parkinson’s disease is a global challenge, but efforts to tackle this must begin at a National level: a protocol for National digital screening and eat, move, sleep lifestyle interventions to prevent or slow the rise of non-communicable diseases in Thailand. Front Neurol. 2024;15:1386608. 38803644 10.3389/fneur.2024.1386608 PMC11129688

[3] Adam H, Gopinath SCB, Md Arshad MK, Adam T, Parmin NA, Husein I, et al. An update on pathogenesis and clinical scenario for Parkinson’s disease: diagnosis and treatment. 3 Biotech. 2023;13(5):142. 37124989 10.1007/s13205-023-03553-8 PMC10134733

[4] Morris HR, Spillantini MG, Sue CM, Williams-Gray CH. The pathogenesis of parkinson’s disease. Lancet. 2024;403(10423):293–304. 38245249 10.1016/S0140-6736(23)01478-2

[5] Bloem BR, Okun MS, Klein C. Parkinson’s disease. Lancet. 2021;397(10291):2284–303. 33848468 10.1016/S0140-6736(21)00218-X

[6] Mohamed YT, Salama A, Rabie MA, Abd El Fattah MA. Neuroprotective effect of Secukinumab against rotenone induced parkinson’s disease in rat model: involvement of IL-17, HMGB-1/TLR4 axis and bdnf/trkb cascade. Int Immunopharmacol. 2023;114:109571. 36527875 10.1016/j.intimp.2022.109571

[7] Qi LF, Liu Y, Liu S, Xiang L, Liu Z, Liu Q, et al. Phillyrin promotes autophagosome formation in A53T-alphaSyn-induced parkinson’s disease model via modulation of REEP1. Phytomedicine. 2024;134:155952. 39178680 10.1016/j.phymed.2024.155952

[8] Espay AJ, Morgante F, Merola A, Fasano A, Marsili L, Fox SH, Bezard E, Picconi B, Calabresi P, Lang AE. Levodopa-induced dyskinesia in Parkinson disease: current and evolving concepts. Ann Neurol. 2018;84(6):797–811. 30357892 10.1002/ana.25364

[9] Tan D, Tseng HHL, Zhong Z, Wang S, Vong CT, Wang Y. Glycyrrhizic acid and its derivatives: promising candidates for the management of type 2 diabetes mellitus and its complications. Int J Mol Sci. 2022. 10.3390/ijms231910988. 36232291 10.3390/ijms231910988 PMC9569462

[10] Zulfugarova P, Zivari-Ghader T, Maharramova S, Ahmadian E, Eftekhari A, Khalilov R, et al. A mechanistic review of pharmacological activities of homeopathic medicine licorice against neural diseases. Front Neurosci. 2023;17:1148258. 36950127 10.3389/fnins.2023.1148258 PMC10025333

[11] Ojha S, Javed H, Azimullah S, Abul Khair SB, Haque ME. Glycyrrhizic acid attenuates neuroinflammation and oxidative stress in rotenone model of Parkinson’s disease. Neurotox Res. 2016;29(2):275–87. 26607911 10.1007/s12640-015-9579-z

[12] Wang XF, Zhou QM, Lu YY, Zhang H, Huang S, Su SB. Glycyrrhetinic acid potently suppresses breast cancer invasion and metastasis by impairing the p38 MAPK-AP1 signaling axis. Expert Opin Ther Targets. 2015;19(5):577–87. 25828376 10.1517/14728222.2015.1012156

[13] Bunnell BA. Adipose tissue-derived mesenchymal stem cells. Cells. 2021. 10.3390/cells10123433. 34943941 10.3390/cells10123433 PMC8700397

[14] Wu H, Fan Y, Zhang M. Advanced progress in the role of adipose-derived mesenchymal stromal/stem cells in the application of central nervous system disorders. Pharmaceutics. 2023. 10.3390/pharmaceutics15112637. 38004615 10.3390/pharmaceutics15112637 PMC10674952

[15] Wu J, Huang S, Yu Y, Lian Q, Liu Y, Dai W, Liu Q, Pan Y, Liu GA, Li K, et al. Human adipose and synovial-derived MSCs synergistically attenuate osteoarthritis by promoting chondrocyte autophagy through FoxO1 signaling. Stem Cell Res Ther. 2024;15(1):261. 39148121 10.1186/s13287-024-03870-6 PMC11328463

[16] Xie X, Song Q, Dai C, Cui S, Tang R, Li S, et al. Clinical safety and efficacy of allogenic human adipose mesenchymal stromal cells-derived exosomes in patients with mild to moderate Alzheimer’s disease: a phase I/II clinical trial. Gen Psychiatr. 2023;36(5):e101143. 37859748 10.1136/gpsych-2023-101143 PMC10582850

[17] Park H, Chang KA. Therapeutic potential of repeated intravenous transplantation of human adipose-derived stem cells in subchronic MPTP-induced Parkinson’s disease mouse model. Int J Mol Sci. 2020. 10.3390/ijms21218129. 33143234 10.3390/ijms21218129 PMC7663651

[18] Chen J, Wei J, Huang Y, Ma Y, Ni J, Li M, Zhu Y, Gao X, Fan G. Danhong injection enhances the therapeutic efficacy of mesenchymal stem cells in myocardial infarction by promoting angiogenesis. Front Physiol. 2018;9:991. 30093864 10.3389/fphys.2018.00991 PMC6070728

[19] Shenzhong J, Han W, Chengxian Y, Feng F, Dan X, Mengyu Z, Manqing X, Ruixue C, Zhaohui Z, Chenhao J et al. Phase 1 study of safety and preliminary efficacy of intranasal transplantation of human neural stem cells (ANGE-S003) in Parkinson’s disease. J Neurol Neurosurg Psychiatry 2024(0). 10.1136/jnnp-2023-332921 38724232

[20] Themistokleous C, Bagnoli E, Parulekar R, Muqit MMK. Role of autophagy pathway in Parkinson’s disease and related genetic neurological disorders. J Mol Biol. 2023;435(12):168144. 37182812 10.1016/j.jmb.2023.168144

[21] Sakurai M, Kuwahara T. Canonical and noncanonical autophagy: involvement in parkinson’s disease. Front Cell Dev Biol. 2025;13:1518991. 39949604 10.3389/fcell.2025.1518991 PMC11821624

[22] Nechushtai L, Frenkel D, Pinkas-Kramarski R. Autophagy in Parkinson’s disease. Biomolecules. 2023. 10.3390/biom13101435. 37892117 10.3390/biom13101435 PMC10604695

[23] Navarro-Romero A, Montpeyo M, Martinez-Vicente M. The emerging role of the lysosome in Parkinson’s disease. Cells. 2020. 10.3390/cells9112399. 33147750 10.3390/cells9112399 PMC7692401

[24] Cai S, Bi Z, Bai Y, Zhang H, Zhai D, Xiao C, Tang Y, Yang L, Zhang X, Li K, et al. Glycyrrhizic Acid-Induced differentiation repressed stemness in hepatocellular carcinoma by targeting c-Jun N-Terminal kinase 1. Front Oncol. 2019;9:1431. 31998631 10.3389/fonc.2019.01431 PMC6962306

[25] Liu P, Xu Y, Ye J, Tan J, Hou J, Wang Y, et al. Qingre huazhuo jiangsuan decoction promotes autophagy by inhibiting PI3K/AKT/mTOR signaling pathway to relieve acute gouty arthritis. J Ethnopharmacol. 2023;302(Pt A):115875. 36328206 10.1016/j.jep.2022.115875

[26] Chen Y, Pan X, Zhao J, Li C, Lin Y, Wang Y, Liu X, Tian M. Icariin alleviates osteoarthritis through PI3K/Akt/mTOR/ULK1 signaling pathway. Eur J Med Res. 2022;27(1):204. 36253872 10.1186/s40001-022-00820-x PMC9575285

[27] Heras-Sandoval D, Perez-Rojas JM, Hernandez-Damian J, Pedraza-Chaverri J. The role of PI3K/AKT/mTOR pathway in the modulation of autophagy and the clearance of protein aggregates in neurodegeneration. Cell Signal. 2014;26(12):2694–701. 25173700 10.1016/j.cellsig.2014.08.019

[28] Lestón Pinilla L, Ugun-Klusek A, Rutella S, De Girolamo LA. Hypoxia signaling in Parkinson’s disease: there is use in asking what HIF? Biology. 2021. 10.3390/biology10080723. 34439955 10.3390/biology10080723 PMC8389254

[29] Fu G, Kang X, Lin S. Glycyrrhizic acid inhibits hippocampal neuron apoptosis by activating the PI3K/ AKT signaling pathway. Biochem Genet 2024. 10.1007/s10528-024-10936-w 39377899

[30] Jin Z, Xiang R, Dai J, Wang Y, Xu Z. HIF-1α mediates CXCR4 transcription to activate the akt/mtor signaling pathway and augment the viability and migration of activated B cell-like diffuse large B-cell lymphoma cells. Mol Carcinog. 2023;62(5):676–84. 36789975 10.1002/mc.23515

[31] Lal R, Singh A, Watts S, Chopra K. Experimental models of Parkinson’s disease: challenges and opportunities. Eur J Pharmacol. 2024;980:176819. 39029778 10.1016/j.ejphar.2024.176819

[32] Gugliandolo A, Bramanti P, Mazzon E. Mesenchymal stem cell therapy in parkinson’s disease animal models. Curr Res Transl Med. 2016;65(2):51–60. 28466824 10.1016/j.retram.2016.10.007

[33] Division of Nephrology DoIMI, Hospital U, Division for Allergy G-U, Adolescents Medicine P et al. UHF, Goethe University, 60596 Frankfurt am Main, Germany., Experimental Immunology DfC, Adolescents Medicine UHF, Goethe University, 60596 Frankfurt am Main, Germany. Tracking of Infused Mesenchymal Stem Cells in Injured Pulmonary Tissue in Atm-Deficient Mice. Cells : 2020, 9(6). 10.3390/cells9061444 PMC7349119 32531978

[34] Choi HS, Kim HJ, Oh J-H, Park H-G, Ra JC, Chang K-A, et al. Therapeutic potentials of human adipose-derived stem cells on the mouse model of parkinson’s disease. Neurobiol Aging. 2015;36(10):2885–92. 26242706 10.1016/j.neurobiolaging.2015.06.022

[35] Biedler JL, Roffler-Tarlov S, Schachner M, Freedman LS. Multiple neurotransmitter synthesis by human neuroblastoma cell lines and clones. Cancer Res. 1978;38(11 Pt 1):3751–7. 29704

[36] Zhou C, Li M, Zhang Y, Ni M, Wang Y, Xu D, Shi Y, Zhang B, Chen Y, Huang Y, et al. Autologous adipose-derived stem cells for the treatment of crohn’s fistula-in-ano: an open-label, controlled trial. Stem Cell Res Ther. 2020;11(1):124. 32183875 10.1186/s13287-020-01636-4 PMC7079384

[37] Zhu M, Heydarkhan-Hagvall S, Hedrick M, Benhaim P, Zuk P. Manual isolation of adipose-derived stem cells from human lipoaspirates. J Vis Exp 2013;(79):e50585. 10.3791/50585 PMC3935774 24121366

[38] Hua G, Xiaolei L, Weiwei Y, Hao W, Yuangang Z, Dongmei L, Yazhuo Z, Hui Y. Protein phosphatase 2A is involved in the tyrosine hydroxylase phosphorylation regulated by α-synuclein. Neurochem Res. 2015;40(3):428–37. 25567480 10.1007/s11064-014-1477-x

[39] Cardo LF, Sandoval JM, Li Z, Webber C, Li M. Single-cell transcriptomics and in vitro lineage tracing reveals differential susceptibility of human iPSC-derived midbrain dopaminergic neurons in a cellular model of parkinson’s disease. Cells. 2023;12(24):2860. 38132179 10.3390/cells12242860 PMC10741976

[40] Tanna T, Sachan V. Mesenchymal stem cells: potential in treatment of neurodegenerative diseases. Curr Stem Cell Res Ther. 2014;9(6):513–21. 25248677 10.2174/1574888x09666140923101110

[41] Tian J, Li X, Zhao L, Shen P, Wang Z, Zhu L, Li C, Su C, Zhang Y. Glycyrrhizic acid promotes neural repair by directly driving functional remyelination. Food Funct. 2020;11(1):992–1005. 31808502 10.1039/c9fo01459d

[42] Helena X, Bé W. The SH-SY5Y cell line in parkinson’s disease research: a systematic review. Mol Neurodegener. 2017;12(1):10. 28118852 10.1186/s13024-017-0149-0 PMC5259880

[43] Eva V, Nina D, Marloes L, Jeannette H, Monica F. Degree of differentiation impacts neurobiological signature and resistance to hypoxia of SH-SY5Y cells. J Neural Eng. 2023;20(6):066038. 10.1088/1741-2552/ad17f3 38128130

[44] Jasmina I, Klara Š, Barbara B, Dinko M. Mesenchymal stem cell therapy for neurological disorders: the light or the dark side of the force? Front Bioeng Biotechnol. 2023;11:1139359–1139359. 36926687 10.3389/fbioe.2023.1139359 PMC10011535

[45] d’Angelo M, Cimini A, Castelli V. Insights into the effects of mesenchymal stem cell-derived secretome in parkinson’s disease. Int J Mol Sci. 2020;21(15):5241. 32718092 10.3390/ijms21155241 PMC7432166

[46] Isabelle ZZE, Xingchi C, Xuegang C. Exosomal microRNAs as novel cell-free therapeutics in tissue engineering and regenerative medicine. Biomedicines. 2022;10(10):2485–2485. 36289747 10.3390/biomedicines10102485 PMC9598823

[47] Hou Y, Chu X, Park JH, Zhu Q, Hussain M, Li Z, et al. Urolithin A improves alzheimer’s disease cognition and restores mitophagy and lysosomal functions. Alzheimers Dement. 2024;20(6):4212–33. 38753870 10.1002/alz.13847 PMC11180933

[48] Chen HX, Liang FC, Gu P, Xu BL, Xu HJ, Wang WT, et al. Exosomes derived from mesenchymal stem cells repair a Parkinson’s disease model by inducing autophagy. Cell Death Dis. 2020;11(4):288. 32341347 10.1038/s41419-020-2473-5 PMC7184757

[49] Yang G, Li J, Cai Y, Yang Z, Li R, Fu W. Glycyrrhizic acid alleviates 6-hydroxydopamine and corticosterone-induced neurotoxicity in SH-SY5Y cells through modulating autophagy. Neurochem Res. 2018;43(10):1914–26. 30206804 10.1007/s11064-018-2609-5

[50] Wang YL, Liu XS, Wang SS, Xue P, Zeng ZL, Yang XP, Zhang SM, Zheng W, Hua L, Li JF, et al. Curcumin-activated mesenchymal stem cells derived from human umbilical cord and their effects on MPTP-mouse model of Parkinson’s disease: a new biological therapy for Parkinson’s disease. Stem Cells Int. 2020;2020:4636397. 32148518 10.1155/2020/4636397 PMC7048946

[51] Wu L-K, Agarwal S, Kuo C-H, Kung Y-L, Day CH, Lin P-Y, Lin S-Z, Hsieh DJ-Y, Huang C-Y, Chiang C-Y. Artemisia Leaf Extract protects against neuron toxicity by TRPML1 activation and promoting autophagy/mitophagy clearance in both in vitro and in vivo models of MPP+/MPTP-induced Parkinson’s disease. Phytomedicine. 2022;104:15420. 10.1016/j.phymed.2022.154250 35752074

[52] Yu L, Hu X, Xu R, Zhao Y, Xiong L, Ai J, Wang X, Chen X, Ba Y, Xing Z, et al. Piperine promotes PI3K/AKT/mTOR-mediated gut-brain autophagy to degrade alpha-Synuclein in parkinson’s disease rats. J Ethnopharmacol. 2024;322:117628. 38158101 10.1016/j.jep.2023.117628

[53] Zhu JH, Horbinski C, Guo F, Watkins S, Uchiyama Y, Chu CT. Regulation of autophagy by extracellular signal-regulated protein kinases during 1-methyl-4-phenylpyridinium-induced cell death. Am J Pathol. 2007;170(1):75–86. 17200184 10.2353/ajpath.2007.060524 PMC1762689

[54] You-Tong W, Hui-Ling T, Guanghou S, Chantal B, Qing H, Choon-Nam RWM, Patrice O, Han-Ming C. Dual role of 3-methyladenine in modulation of autophagy via different Temporal patterns of Inhibition on class I and III phosphoinositide 3-kinase. J Biol Chem. 2010;285(14):10850–61. 20123989 10.1074/jbc.M109.080796 PMC2856291

[55] Saxton RA, Sabatini DM. mTOR signaling in growth, metabolism, and disease. Cell. 2017;168(6):960–76. 28283069 10.1016/j.cell.2017.02.004 PMC5394987

[56] Li R, Zheng Y, Zhang J, Zhou Y, Fan X. Gomisin N attenuated cerebral ischemia-reperfusion injury through inhibition of autophagy by activating the PI3K/AKT/mTOR pathway. Phytomedicine. 2023;110:154644. 36634381 10.1016/j.phymed.2023.154644

