# Supplementary material for: Glycyrrhizic acid combined with human adipose-derived MSCs synergistically alleviates the MPP+/MPTP-induced parkinson’s disease by inducing autophagy through PI3K/AKT/HIF-1α pathway
Source: Stem Cell Res Ther. 2025 Sep 25;16:500. doi: 10.1186/s13287-025-04626-6 (PMC12465595; doi:10.1186/s13287-025-04626-6)
Supplement: Supplementary file 1 — Supplementary material 1. [file 13287_2025_4626_MOESM1_ESM.docx]

**Supplementary Material**

**
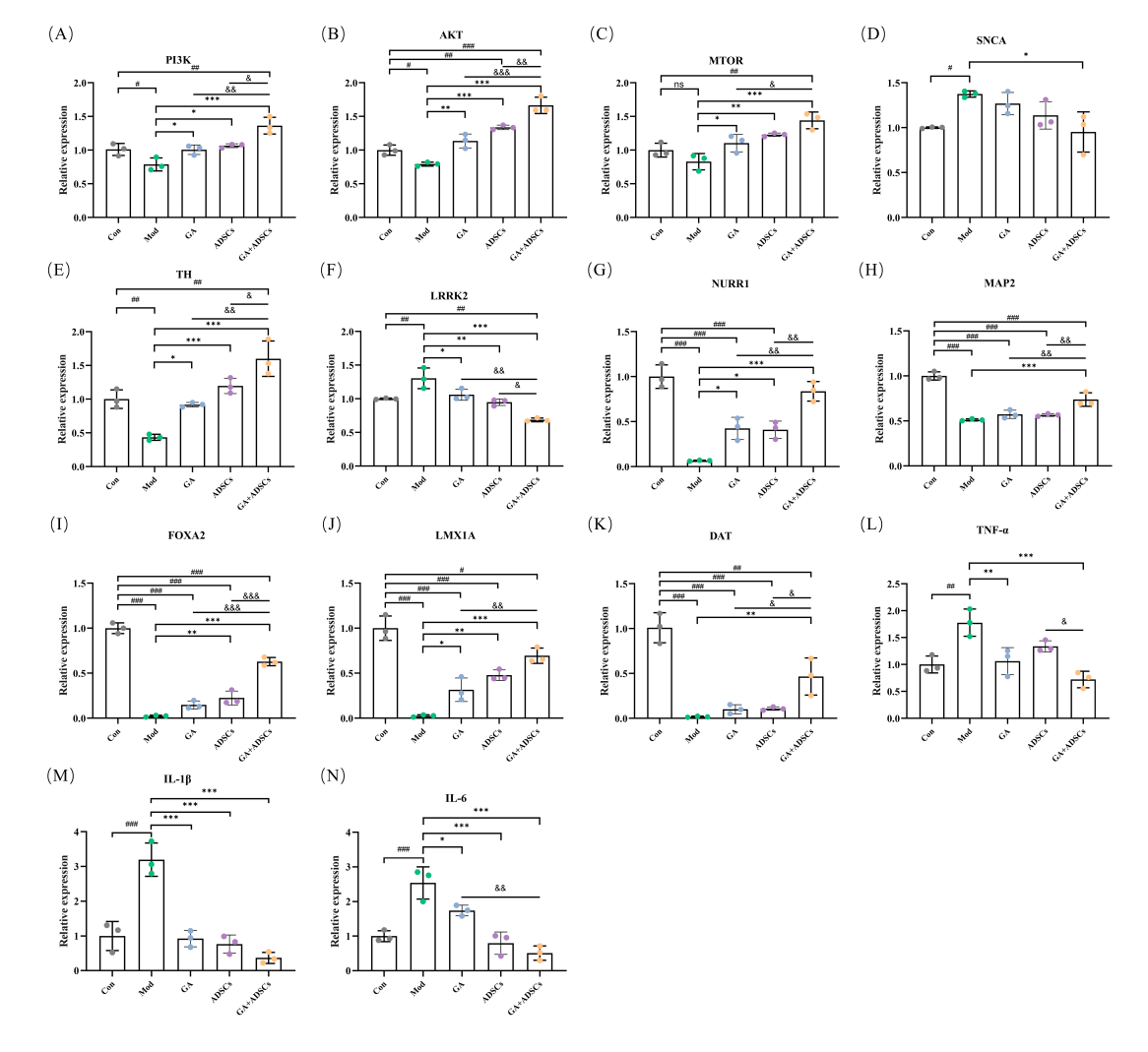
**

**S1 Fig. Representative expression of *PI3K* (A), *AKT* (B), *mTOR* (C), *SNCA* (D), *TH* (E), *LRRK2* (F), *NURR1* (G), *MAP2* (H), *FOXA2* (I), *LMX1A* (J), *DAT* (K), *TNF-α* (L), *IL-1β* (M), and *IL-6* (N) detected by qRT-PCR.** n = 3. ^#^p < 0.05, ^##^p < 0.01 and ^###^p< 0.001 vs. Con; ^*^p < 0.05, ^**^p < 0.01 and ^***^p < 0.001 vs. Mod; ^&^p < 0.05, ^&&^p < 0.01 and ^&&&^p < 0.001 vs. GA+ADSCs. Data are expressed as mean ± SD.


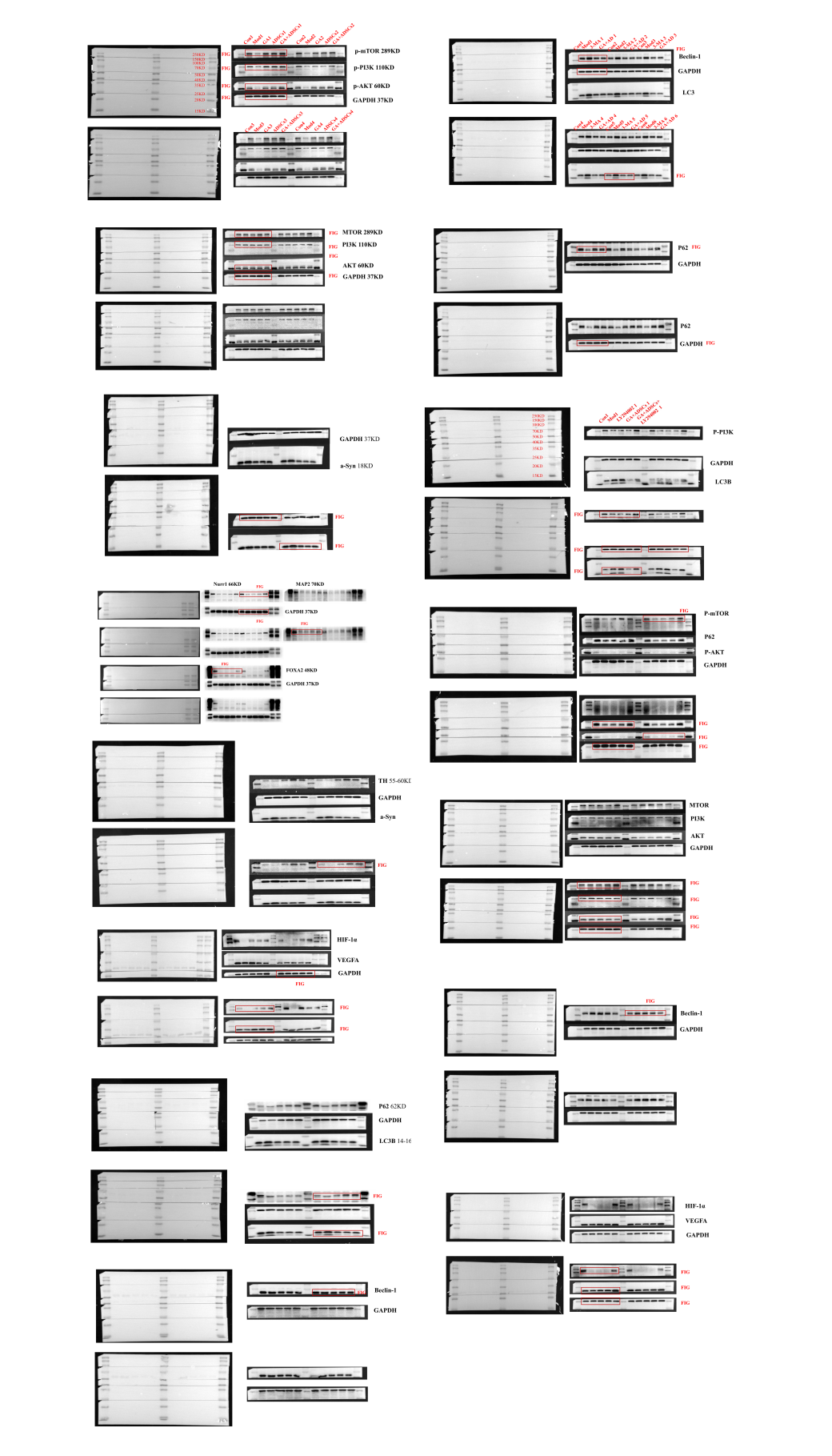


**S2 Fig. Original strip diagrams of western blot.**

|  | Con | Mod | GA | ADSCs | GA+ADSCs |
| --- | --- | --- | --- | --- | --- |
| Upper chamber (ADSCs) | / | / | / | 2×10^5^ | 2×10^5^ |
| Upper chamber (GA) | / | / | / | / | 1.5ml |
| Lower chamber (SH-SY5Y cell) | 2×10^6^ | 2×10^6^ | 2×10^6^ | 2×10^6^ | 2×10^6^ |
| Lower chamber (GA) | / | / | 2.5ml | / | 2.5ml |

**S1 Table. SH-SY5Y, ADSCs, and GA coculture ratio and cell counts.**

| **Antibodies** | **Sources** | **Identifier** |
| --- | --- | --- |
| Rabbit monoclonal to TH | GeneTex | GTX10372 |
| Rabbit monoclonal to α-Syn | Abcam | ab212184 |
| Rabbit polyclonal to p-PI3K | Abmart | T40116 |
| Rabbit monoclonal to PI3K | CellSignaling Technology | 4257 |
| Rabbit polyclonal to p-AKT | GeneTex | GTX128414 |
| Rabbit monoclonal to AKT | Abcam | ab81283 |
| Rabbit monoclonal to p-mTOR | CellSignaling Technology | 5536 |
| Rabbit monoclonal to mTOR | Abcam | ab134903 |
| Rabbit monoclonal to HIF-1α | Abcam | ab179483 |
| Rabbit monoclonal to VEGFA | Abcam | ab214424 |
| Rabbit monoclonal to LC3B | Abcam | ab192890 |
| Rabbit monoclonal to Beclin-1 | Abcam | ab207612 |
| Rabbit monoclonal to P62 | GeneTex | GTX636328 |
| Rabbit polyclonal to MAP2 | Proteintech | 17490-1-AP |
| Rabbit polyclonal to Nurr1 | Proteintech | 10975-2-AP |
| Rabbit polyclonal to FOXA2 | Proteintech | 22474-1-AP |
| Mouse monoclonal to TH | CellSignaling Technology | 45648 |
| Rabbit monoclonal to GAPDH | CellSignaling Technology | 2118 |
| Anti-rabbit IgG, HRP  -linked Antibody | CellSignaling Technology | 7074 |

**S2 Table. Primary and secondary antibodies.**
